# Physiological and transcriptomic responses in the seed coat of field-grown soybean (*Glycine max* L. Merr.) to abiotic stress

**DOI:** 10.1186/s12870-017-1188-y

**Published:** 2017-12-12

**Authors:** Courtney P. Leisner, Craig R. Yendrek, Elizabeth A. Ainsworth

**Affiliations:** 1 Department of Plant Biology, University of Illinois, Urbana-Champaign, Urbana, IL 61801 USA; 2 Institute for Genomic Biology, University of Illinois, Urbana-Champaign, Urbana, IL 61801 USA; 3 USDA ARS Global Change and Photosynthesis Research Unit, 1201 W Gregory Drive, Urbana, IL 61801 USA; 4 Current address: Department of Plant Biology, Michigan State University, East Lansing, MI 48824 USA; 5 Current address: The Scotts Company, Marysville, OH 43040 USA

**Keywords:** Climate change, Seed coat, *Glycine max*, Transcriptome, Sink strength, DNA replication

## Abstract

**Background:**

Understanding how intensification of abiotic stress due to global climate change affects crop yields is important for continued agricultural productivity. Coupling genomic technologies with physiological crop responses in a dynamic field environment is an effective approach to dissect the mechanisms underpinning crop responses to abiotic stress. Soybean (*Glycine max* L. Merr. cv. Pioneer 93B15) was grown in natural production environments with projected changes to environmental conditions predicted for the end of the century, including decreased precipitation, increased tropospheric ozone concentrations ([O_3_]), or increased temperature.

**Results:**

All three environmental stresses significantly decreased leaf-level photosynthesis and stomatal conductance, leading to significant losses in seed yield. This was driven by a significant decrease in the number of pods per node for all abiotic stress treatments. To understand the underlying transcriptomic response involved in the yield response to environmental stress, RNA-Sequencing analysis was performed on the soybean seed coat, a tissue that plays an essential role in regulating carbon and nitrogen transport to developing seeds. Gene expression analysis revealed 49, 148 and 1,576 differentially expressed genes in the soybean seed coat in response to drought, elevated [O_3_] and elevated temperature, respectively.

**Conclusions:**

Elevated [O_3_] and drought did not elicit substantive transcriptional changes in the soybean seed coat. However, this may be due to the timing of sampling and does not preclude impacts of those stresses on different tissues or different stages in seed coat development. Expression of genes involved in DNA replication and metabolic processes were enriched in the seed coat under high temperate stress, suggesting that the timing of events that are important for cell division and proper seed development were altered in a stressful growth environment.

**Electronic supplementary material:**

The online version of this article (10.1186/s12870-017-1188-y) contains supplementary material, which is available to authorized users.

## Background

Rising global temperature, drought stress and increased exposure to air pollutants have contributed to decreased regional and global crop production [1–3] and represent a challenge for future agriculture [4, 5]. If fossil fuel emissions continue at their current pace, global land surface temperatures are projected to increase by 5–9 °C by the end of the century [6]. Increased demand for soil moisture imposed by higher temperatures, coupled with climate projections of more variable precipitation patterns in the future, will result in increased drought stress [7, 8]. Higher temperatures will also favor formation of atmospheric pollutants, including ozone (O_3_), which significantly decreases plant productivity [9].

Genomic approaches have proven powerful tools to understand the complex relationship among genes, proteins and metabolites involved in plant responses to abiotic stress and future climate change [10–14]. In addition, there is growing awareness of the importance of investigating the mechanisms of crop response to environmental change in the dynamic field environment where multiple variables interact [15–23]. Coupling integrated analyses at the molecular, biochemical, physiological and agronomic level of crop responses to global climate change within a production environment has led to better understanding of the underlying transcriptomic responses responsible for complex phenotypes observed across plant species and stressors [24–26].

Soybean (*Glycine max* L. Merr.) is the most widely grown legume worldwide and provides an important global source of oil and protein for food and feed [27]. Soybean seeds provide the economic value for the commodity and have been used as a model system for identifying genes and gene networks required for seed development [28, 29]. The soybean seed coat is a critical tissue that serves as a conduit for water and nutrients [30, 31], coordinates embryo and endosperm growth [32], and encapsulates and protects the embryo at maturity [33]. As the link between maternal and filial tissue, the seed coat plays a critical role in the metabolic control of seed development, and in turn, successful seed production [34]. This is achieved, in part, by the activities of acid invertases (vacuolar and cell wall) and sucrose synthase, which facilitate sucrose transport by generating a strong sucrose-to-hexose gradient across the apoplastic space between the seed coat and the developing seed [30, 35–37]. It has been demonstrated that high temperature or drought stress imposed during soybean seed development can cause changes in seed coat morphology leading to negative effects on seed quality, seed germination rate, and seedling vigor [38–40].

Global transcriptional profiling studies have described the genetic events involved in soybean seed development [28, 29, 41–43], seed coat pigment color [44] and identified a complex seed coat specific transcriptome [31]. However, the effects of abiotic stress on gene expression patterns in the seed coat have not been explored. Here, we investigated the transcriptional response of soybean seed coat tissue to abiotic stresses including drought, elevated O_3_ concentration ([O_3_]), or elevated temperature throughout the growing season in a field setting. Due to the critical role the seed coat plays in supplying nutrients to developing seeds, we investigated abiotic stress-mediated transcriptional changes during the pod filling stage and coupled this with physiological and biochemical activity to identify genes involved in abiotic stress response in the seed coat.

## Results

### Photosynthesis, but not photoassimilate transport to the seed coat, is altered by abiotic stress in soybean

**Fig. 1:**
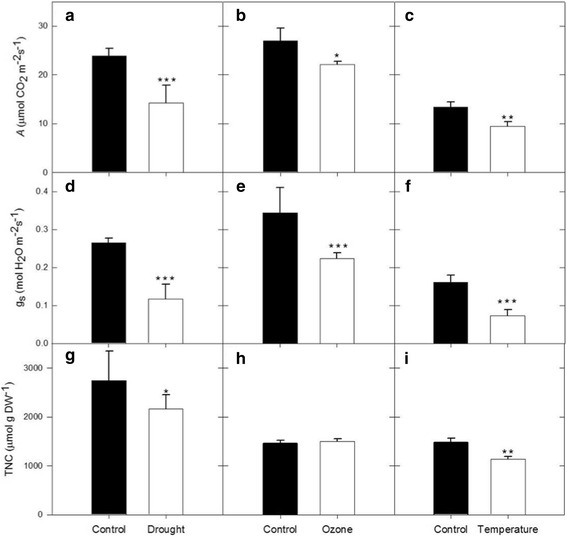
The effects of abiotic stress on primary metabolism. (**a**-**c**) Carbon assimilation rate (*A*), (**d**-**f**) stomatal conductance (*g*_*s*,_) and (**g**-**i**) foliar total non-structural carbohydrate content (TNC) of soybeans grown in season-long drought (**a**, **d**, **g**), elevated [O_3_] (**b**, **e**, **h**) and elevated temperature (**c**, **f**, **i**) conditions. *A* and *g*_*s*_ measurements were taken on different days for ozone and temperature treatments and different year for drought treatment. This was done in order to capture the proper development stage. The light level for *A* and *g*_*s*_ measurements for the drought treatment was 1900 μmol m^−2^ s^−1 ^, 1650 μmol m^−2^ s^−1^ for the ozone treatment, and 450 μmol m^−2^ s^−1^ for the temperature treatment (due to cloudy day conditions). Asterisks represent significance (* *p* < 0.05, ** *p* < 0.01, *** *p* < 0.001)

Leaf-level photosynthetic and biochemical measurements were taken to characterize the effects of abiotic stress on field-grown soybean. Soybeans exposed to drought, elevated [O_3_], or elevated temperature had significantly lower rates of photosynthesis (*A*) and stomatal conductance (*g*_s_; Fig. 1a-f; *p*-value <0.10). *A* was reduced by 20–40% with exposure to abiotic stress, while *g*_s_ was reduced by 35–56% (Fig. 1a-f). Leaf total nonstructural carbohydrate (TNC) content was also significantly decreased in the elevated temperature (Fig. 1i, *p*-value <0.10) and drought (Fig. 1g, *p*-value <0.10) treatments, but not in elevated [O_3_] (Fig. 1h, *p*-value >0.10). Average leaf TNC values for elevated temperature and drought were 1131.9 and 2166.0 μmol g DW^−1^, respectively, representing a 23.1 and 21.2% decrease from control conditions.

**Fig. 2:**
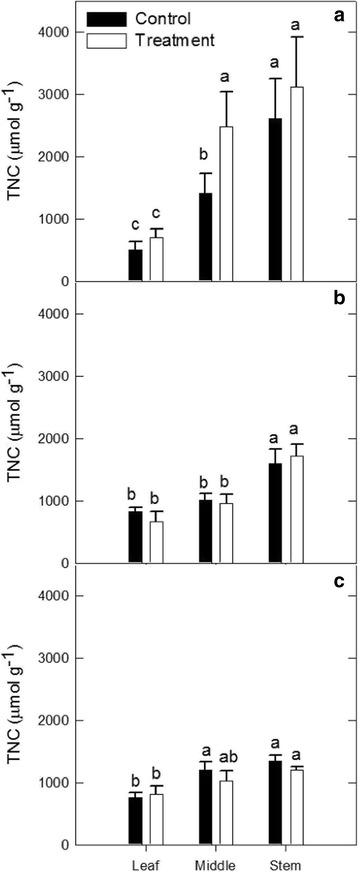
Carbohydrate gradient along the leaf petiole. TNC content of the petiole lengths at three positions along the petiole (leaf adjacent, middle and stem adjacent) for (**a**) drought, (**b**) elevated [O_3_] and (**c**) elevated temperature. Different letters represent significant differences at *p* < 0.10

**Fig. 3:**
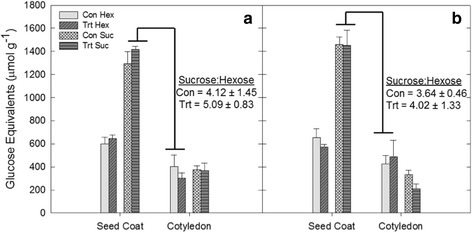
Maintenance of sink strength during pod-fill. Hexose and sucrose content in the seed coat and cotyledon tissues for (**a**) ambient and elevated [O_3_], and (**b**) ambient and elevated temperature. Sucrose:hexose was calculated as the ratio between seed coat sucrose and cotyledon hexose content and is not significantly different for either stress treatment. Con Hex: control hexose concentration; Trt Hex: treatment hexose concentration; Con Suc: control sucrose concentration; Trt Suc: treatment sucrose concentration

The abundance of TNCs was also quantified at three positions along the petiole based on the proximity to source and sink tissues (Fig. 2). There was a significant difference between petiole TNC at the leaf position (source) and stem position (sink) for all treatments (Fig. 2a-c, *p*-value <0.10). These differences were likely driven by changes in starch content, as sucrose content did not differ in different positions of the petiole (data not shown). No significant effect of drought, elevated [O_3_], or elevated temperature on petiole TNC was observed at any position (Fig. 2a-c) (*p*-value >0.10), with the exception of the middle position in the drought treatment, which saw increased petiole TNC in drought conditions (Fig. 2a; *p*-value <0.10). Additionally, a similar high ratio of seed coat sucrose to cotyledon hexose was maintained in the seeds of plants grown at elevated [O_3_] and elevated temperature (Fig. 3) suggesting that sink strength at the seed coat was not altered by those stresses [35]. The average ratios of seed coat sucrose to cotyledon hexose concentration in elevated [O_3_] and elevated temperature conditions were 5.09 and 4.02, which were not significantly different from the ratios in ambient conditions (Fig. 3; *p*-value >0.10).

At maturity, the number of pods per node was measured in all experimental plots and total seed yield was measured in the drought and elevated temperature plots (Table 1). The total number of pods per node decreased for all abiotic stress treatments. There were 3.3–3.4 pods per node in ambient conditions, and growth under abiotic stress conditions reduced pod number to 2.7–3.0 pods per node (Table 1). Seed yield was also reduced by 25.4% in drought and 20.5% in elevated temperature (Table 1).

**Table 1:** Whole plot seed yield and number of pods per node (PPN) in response to three abiotic stress treatments and their respective control

Treatment	Ambient Seed Yield (g m^−2^)	Treatment Seed Yield (g m^−2^)	% Yield Decrease	Ambient PPN	Treatment PPN	PPN *p*-value	PPN d.f.	Optimal α	Optimal β
*Drought*	337.7 ± 15.9	251.8 ± 34.0	25.4	3.4 ± 0.3	2.7 ± 0.1	0.181‡	2	0.38	0.40
*Elevated [O*_*3*_ *]*	–	–	–	3.4 ± 0.2	2.8 ± 0.2	0.120‡	3	0.33	0.39
*Elevated Temperature*	373.6 ± 15.2	297.2 ± 17.9	20.5	3.3 ± 0.2	3.0 ± 0.1	0.144‡	3	0.33	0.39

Values represent the mean ± the standard error

PPN = pods per node; d.f. = degrees of freedom from each pod per node data

‡ = significant based on optimal α analysis (See Methods section)

### Analysis of global changes in expression abundance across multiple abiotic stress conditions in the soybean seed coat

**Fig. 4:**
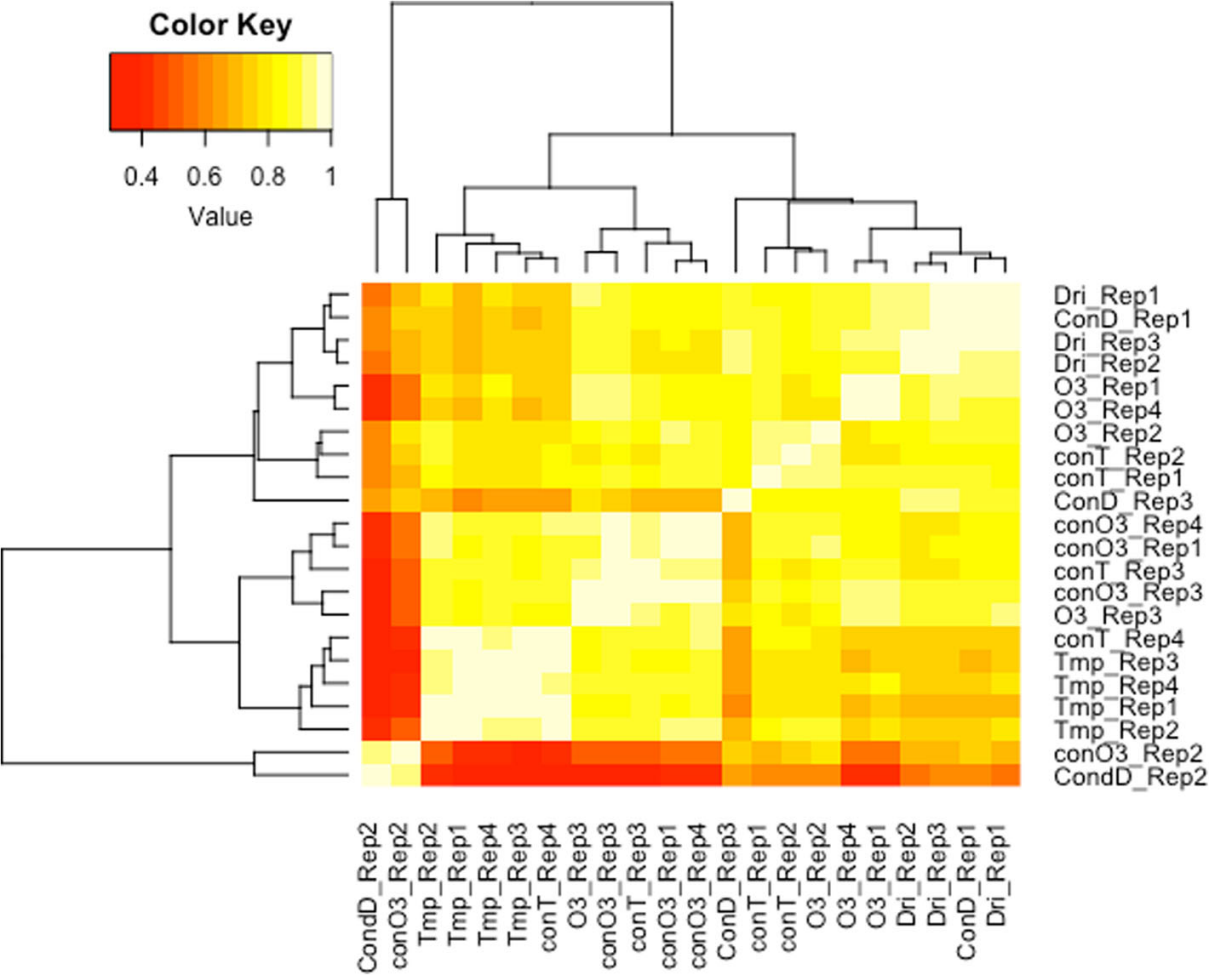
Heat map of Pearson correlation coefficient values across all biological replicates and treatments. ConD: drought control; ConO3: ozone control; ConT: temperature control; Dri: drought; O3: elevated ozone, Tmp: elevated temperature

The quality of all RNA-Sequencing (RNA-Seq) libraries was assessed based on mapping alignment statistics (Additional file 1: Table S1). All libraries except two aligned at >80% to the reference genome. Analysis of the low mapping libraries showed some contamination with bean pod mottle virus (drought treatment replicate 1) and various non-plant contaminations (drought treatment replicate 2). Pearson correlation coefficients were calculated between all biological replicates per treatment. A high correlation was expected between replicates (per treatment), and replicates with low R^2^ values <0.60 were removed from the analysis (Fig. 4) [45]. Hierarchical clustering of expression data demonstrated stronger clustering of the temperature treatment samples, compared to drought and elevated [O_3_] treatment samples (Fig. 4).

Differential gene expression analysis revealed relatively few differentially expressed genes in the soybean seed coat in response to drought and elevated [O_3_], as may have been predicted from the clustering analysis. Only 49 differentially expressed genes in the soybean seed coat were detected in response to drought, and 148 differentially expressed genes in response to elevated [O_3_] (FDR *p*-value <0.10) (Additional file 2: Table S2). In contrast, 1576 differentially expressed genes were detected in the soybean seed coat in response to elevated temperature (Additional file 2: Table S2). However, as we only sampled a single time point during soybean pod fill (R5), we cannot rule out that elevated [O_3_] and drought elicit more substantive transcriptional changes in other tissues, or at different developmental time points. Gene ontology (GO) term analysis did not reveal any enriched terms in the drought and elevated [O_3_] treatments; therefore, we focused our transcriptional analysis on the elevated temperature treatment.

**Table 2:** Minichromosome maintenance (MCM) genes with increased expression under elevated temperature

Gene	LogFC	Average FPKM Elevated Temperature	Functional Annotation
Glyma03g37770	0.93	11.33	MCM7
Glyma05g25980	0.98	8.90	MCM3
Glyma07g36680	0.90	18.91	MCM2
Glyma08g08920	0.70	8.79	MCM3
Glyma09g05240	1.00	10.49	MCM6
Glyma11g12110	1.03	14.38	MCM4
Glyma12g04320	0.94	5.97	MCM4
Glyma13g22420	0.93	11.96	MCM5
Glyma15g16570	0.72	10.67	MCM6
Glyma17g03920	0.84	11.06	MCM2
Glyma17g11220	1.06	10.07	MCM5
Glyma19g40370	1.05	12.56	MCM7

LogFC = log fold-change, FPKM = Fragment per Kilobase per Million Mapped reads

Genes that showed the largest increase in expression (log fold-change) in the elevated temperature treatment included peroxidase proteins, sugar transporter proteins, MYB-domain and leucine-rich repeat domain proteins, and long-chain-alcohol oxidase proteins (Additional file 2: Table S2). Additionally, genes with the highest overall expression in the elevated temperature treatment, represented as the normalized fragment per kilobase of transcript per million mapped reads (FPKM), included BURP domain-containing proteins, peroxidase family proteins, extension-like proteins, senescence-associated genes, and seed storage albumin superfamily proteins (Additional file 3: Table S3).

Differentially expressed genes in elevated temperature were related to chlorophyll biosynthetic processes, DNA replication, and nucleosome assembly, based on GO analysis (Additional file 4: Figure S1). Functional analysis of genes with GO terms related to DNA replication identified twelve minichromosome maintenance (MCM) family protein genes (Table 2). MCM proteins are licensing factors and part of the pre-replicative complex (pre-RC) in eukaryotes, playing an essential role in cell division [46]. *MCM2* to *MCM7* encode subunits of the MCM(2–7) hexamer helicase that is recruited to replication origins [47]. In our study, two genes from each of the MCM families 2 through 7 were significantly increased by growth at elevated temperature. To further understand the role of MCM genes in response to elevated temperature in the soybean seed coat additional analyses were completed.

### MCM genes may play a role in maintaining proper DNA replication in the soybean seed coat under elevated temperature stress

Multiple sequence alignment of all twelve differentially expressed MCM genes in the soybean seed coat was completed with the sequences of known MCM genes in Arabidopsis (AtMCM2–7), maize (ZmMCM2–7), pea (PsMCM2–7), and two *Brassica* species (*B. oleracea* BoMCM2–7, *B. rapa* BrMCM2–7) (Additional file 5: Figure S2). Soybean MCM2 and MCM7 genes had between ~76–84% amino acid identity with both *B. oleracea* and *B. rapa* MCM genes, while soybean MCM4, 5 and 6 genes showed higher amino acid sequence similarity with *B. rapa* MCM genes (Additional file 6: Table S4), ranging from ~73–78% identity. Soybean MCM3 (GmMCM3) genes Glyma05g25980 and Glyma08g08920 had 76–77% identity with BoMCM3 and BrMCM3_2, but 32% identity with BrMCM3_1. Soybean MCM6 (GmMCM6) genes Glyma09g05240 and Glyma15g16570 had 75.0 and 74.7% amino acid identity with ZmMCM6, and 84.1 and 84.7% amino acid identity with PsMCM6 (Additional file 6: Table S4). Both GmMCM2 genes Glyma07g36680 and Glyma17g03920 had ~76% amino acid identity with AtMCM2 (Additional file 6: Table S4). The high sequence similarity with known MCM genes in Arabidopsis, maize, pea and *Brassica* species may suggest a similar role of soybean MCM genes in proper development under abiotic stress. Additionally, analysis of expression of these twelve MCM genes in the soybean expression atlas (https://soybase.org) found high expression in young leaf tissue (Additional file 7: Table S5). This indicates that the MCM expression in the soybean seed coat is not unique to that tissue, most likely due to their general role in DNA replication.

## Discussion

Reductions in primary metabolism and yield are common responses to abiotic stress [21, 22, 48–51], and our study confirmed that drought, elevated [O_3_] and elevated temperature reduce photosynthesis and seed yield. We also predicted that photoassimilate available for translocation to developing reproductive tissues would be diminished. However, despite decreased leaf-level CO_2_ assimilation in soybeans grown under drought, elevated [O_3_], and elevated temperature treatments (Fig. 1), translocation of photoassimilate was not altered. Additionally, a high sucrose:hexose ratio between the seed coat and cotyledon was maintained (Fig. 2), which is one possible indicator that the sink strength of individual seeds was not affected by abiotic stress treatments [35], despite a net reduction in sink strength at the whole plant level. To further understand the effects of abiotic stresses on the soybean seed coat, we performed transcriptomic analysis.

Differential gene expression analysis revealed far fewer genes differentially expressed in the drought and elevated [O_3_] treatments compared to the elevated temperature treatment (Additional file 2: Table S2). This suggests that different abiotic stresses do not elicit common transcriptional responses in the soybean seed coat, which supports other transcriptional and metabolomic experiments done with Arabidopsis [52]. However, our analysis was limited to a single time point during the pod filling stage (R5), and it cannot be ruled out that drought and elevated [O_3_] induce more substantive transcriptional responses at other development time points, or in tissues other than the seed coat.

Genes involved with DNA replication showed increased expression in seed coats of soybeans exposed to elevated temperature. In particular, twelve MCM family genes showed greater expression at elevated temperature. MCM proteins form a heterohexomeric complex (MCM2–7) that is a key part of the initiation and elongation steps in eukaryotic DNA replication [46, 47]. MCM proteins also ensure DNA replication occurs only once during the S phase of the cell cycle [53]. How MCM genes control DNA replication, however, is less well understood in plants [46, 47, 54–56]. Previous work in Arabidopsis and maize has found MCM genes are preferentially expressed in young tissues with large numbers of replicating cells [57–63], and in Arabidopsis MCM subunits are coordinately expressed across tissue types and development [54]. MCM proteins are also critical components of plant reproductive development. Work done in Arabidopsis has identified MCM genes essential for embryo development [64], and MCM proteins required for proper cytokinesis during seed development [57, 58, 61]. Work done in maize found ZmMCM6 is an essential protein for both vegetative and reproductive growth [63], and transgenic maize plants with minor antisense transcript amounts of *ZmMCM6* had an overall reduced size and were unable to develop cobs to maturity [63]. We found high sequence similarity with known MCM genes in Arabidopsis, maize, pea and two *Brassica* species (Additional file 6: Table S4), which may indicate a similar functional role of MCM genes in the soybean seed coat.

There is growing evidence that MCM proteins also play a role in plant response to abiotic stress. For example, work in pea (*P. sativum*) has shown MCM6 is associated with salinity tolerance [46]. Furthermore, constitutive expression of *PsMCM6* in tobacco seedlings increased salinity tolerance. Findings from Dang et al. [46] indicate that MCM proteins may interact with proteins that are related to stress tolerance, and/or are involved in transcriptional regulation of stress response genes through their function as helicases. Recently, it was demonstrated that different MCM family genes were up-regulated in *B. oleracea* and *B. rapa* in response to cold and salt stress, suggesting some degree of species-specific response [65]. These previous analyses have suggested that subunits of the MCM complex are not changing in concert in response to stress, and that perhaps different subunits of the MCM complex can respond independently. In our study of soybean seed coats, two transcripts from each of the MCM families 2 through 7 increased expression in elevated temperature stress (Additional file 2: Table S2). We hypothesize that the coordinated increase in expression allowed for greater DNA replication and cell cycle activity mediated by the MCM(2–7) helicase in the soybean seed coat under high temperature stress, possibly due to acceleration of seed development in the temperature stress conditions.

## Conclusions

This study investigated the transcriptomic response of the soybean seed coat to multiple climate change factors in a field environment. Soybean plants exposed to drought, elevated [O_3_], and elevated temperature showed decreased carbon assimilation and stomatal conductance, leading to decreased leaf TNC in drought and elevated temperature treatments. At maturity, soybean yield was also decreased in drought and elevated temperature. While decreased carbon assimilation was observed, there was no observed decrease in photoassimilate transport from source to sink tissue, as measured by petiole TNC abundance at three positions along the petiole. Additionally, sink strength was maintained in the soybean seed coat; a high seed coat sucrose-to-cotyledon hexose ratio was maintained in the soybean seed coat exposed to drought, elevated [O_3_] and elevated temperature. Transcriptomic analysis found elevated temperature caused increased expression of genes related to DNA replication, cell cycle and microtubule motor family proteins, in particular MCM genes. This indicates greater cell cycle and DNA replication activity in seeds exposed to elevated temperature, and represents a possible acceleration of the completion of seed development due to elevated temperature stress.

## Methods

### Experimental site and plant growth conditions

Soybean (*Glycine max* cv. Pioneer 93B15) was grown in drought conditions (*n* = 3) at the 32-hectacre Soybean Free Air Concentration Enrichment (SoyFACE; https://soyface.illinois.edu) experimental field site in the summer of 2011 and in elevated [O_3_] (*n* = 4) and elevated temperature conditions (n = 4) in the summer of 2012. Soybeans were planted on 8 June 2011 and 15 May 2012, at 0.38 m row spacing. Soybean and maize (*Zea mays*) are rotated each year at the experimental facility, and the soybean crop was not fertilized or irrigated. For each stress, soybean plants were grown in control and treatment plots nested within the 32 ha field. Each ozone plot was 21 m in diameter, with control and treatment plots separated by minimum of 100 m. The elevated [O_3_] fumigation system described in [66] increased [O_3_] to 100 nL L^−1^ from ~10:00 to 17:00, except when leaves were wet. In 2012, the season-long 8-h average ambient [O_3_] was 50.6 nL L^−1^_,_ and the 8-h season-long elevated [O_3_] was 69.7 ± 1.3 nL L^−1^. Drought was established by employing modified Solair motorized retractable fabric awnings (Glen Raven, Inc., Glen Raven, NC, http://www.glenraven.com) mounted 25–50 cm above the plant canopy to intercept nighttime rainfall (described in [67]), resulting in a 35% reduction in total growing season precipitation (control precipitation, 274 mm; reduced precipitation, 179 mm). The drought plots were 8 m long and 4 m wide. The elevated temperature treatment was produced using infrared heaters (Salamander Aluminum Extrusion Reflector Assembly Housing for Ceramic Infrared Heaters; Mor Electric Heating Assoc., http://www.morelectricheating.com) fitted with four heating elements (Mor-FTE 1000-W, 240-V heaters; Mor Electric Heating Assoc., http://www.morelectricheating.com) mounted 1.2 m above the plant canopy (described in [21]). The growing season mean increase in temperature was 2.71 °C ± 0.4 °C in the temperature plots.

### Photosynthetic gas exchange, tissue sampling, biochemical analyses and harvest

Gas exchange measurements were taken at mid-day using the middle trifoliate of fully expanded leaves at the 5th node down from the shoot apex during the pod filling stage (R5). This stage is characterized by nutrient accumulation and synthesis of storage proteins [42]. A portable infrared gas analyzer (LI-6400; Licor Biosciences, Inc., Lincoln, NE, http://www.licor.com) was used to take measurements of leaf photosynthesis (*A*) and stomatal conductance (*g*_*s*_) by setting the chamber conditions to reflect the ambient light intensity, temperature and relative humidity in the field. Three leaves from different plants were measured for each treatment and control plot.

Following gas exchange measurements, tissue was collected from the 5th node at dusk (approximately 18:00–20:00) for carbohydrate and gene expression measurements. Leaf discs (1.34 cm^2^) were excised from fully expanded leaves, flash-frozen in liquid N, and then stored at −80 °C. Leaf discs were also collected and dried at 55 °C for one week to assess specific leaf weight. Petioles were removed and sectioned into 2.0 cm lengths based on proximity to the leaf, the stem and at a distance mid-way between the leaf and stem. The seed coat was harvested from detached pods by making a small incision into the seed coat of the seed with a scalpel and separating the seed coat from the cotyledons. Seed coat tissue was collected from several pods per plant in order to fill a 2 mL tube. Seed coats were flash-frozen in liquid N and stored at −80 °C. Seed coat tissue was collected from ~10–20 plants per plot and pooled in order to obtain sufficient tissue for subsequent analyses.

Total non-structural carbohydrate content was calculated from sequential determination of glucose, fructose and sucrose content using the methods of [68]. The pellets remaining after the ethanol extraction were then solubilized by heating to 95 °C in 0.1 M NaOH for subsequent determination of starch content. The NaOH solution was acidified to pH 4.9 and starch content was determined from glucose equivalents [69].

Seed yield from whole-plots was determined at maturity (R8) in the drought and elevated temperature treatments. Seed yield for ambient and treatment plots was measured as total seed weight per area (g m^−2^). At maturity (R8), the number of pods per node was counted at the same node where physiological measurements were made. In 2011 the drought yield was obtained by harvesting all plants within 4, 1 m rows per plot, giving a sampling area of 1.542 m^2^. In 2012, the elevated temperature yield was obtained by harvesting all plants inside a single 1 m row per plot, giving a sampling area of 0.762 m^2^. Final yield for the elevated [O_3_] plots was not measured in 2012.

### Statistical analysis of physiological and biochemical data

All model assumptions of normality and homogeneous error (NID, 0, σ^2^) were examined for each parameter (*A*, *g*_*s*_, TNC, and hexose/sucrose concentrations). Assumptions of normality were tested using the Shapiro-Wilk test, and the assumption of homogeneous variance was examined by plotting the residual versus the predicted value for each variable. A linear mixed model was used to assess the impact of the fixed effect of treatment (drought, elevated [O_3_], or elevated temperature) compared to the control with block as a random factor in the model. The dependent variables leaf TNC, and seed hexose and sucrose concentrations were fit separately. A repeated measures analysis was used for petiole TNC data, due to the correlation in space. For yield data analysis, optimal α values for the pod per node data were analyzed according to [70]. This method minimizes the average of Type I and Type II errors, therefore minimizing the *overall* error rate. This method avoids unnecessarily high rates of Type II error and is appropriate in studies where Type I and Type II errors are considered to have equal importance [70]. Degrees of freedom were taken from each data set (drought, elevated [O_3_] and elevated temperature) and Cohen’s ƒ^2^ were input into R (ver. 3.0.2; www.r-project.org) using code provided by [70]. Cohen’s ƒ^2^ of 0.35 was chosen a priori based on previous literature [71, 72]. Based on the degrees of freedom from each data set the traditional optimal α value to analyze the pod per node data was higher than the standard α = 0.05. All analyses were conducted in SAS (SAS Institute, Version 9.3, Cary, NC, http://www.sas.com/).

### RNA extraction and library preparation

Total RNA was isolated from each biological replicate of frozen seed coat tissue (pooled from multiple plants) following standard protocols. Briefly, seed coats were ground to a fine powder in liquid N using a mortar and pestle. RNA was extracted using the PureLink Plant RNA Reagent (Ambion, by Life Technologies Corp., Grand Island, NY, USA, http://www.lifetechnologies.com) and genomic DNA contamination was removed from RNA samples using TurboDNase treatment (Applied Biosystems by Life Technologies, Austin, TX, USA, http://www.lifetechnologies.com) according to the manufacturer’s protocols. RNA quantity was determined with a spectrophotometer (Nanodrop 1000, Thermo Fisher Scientific, Waltham, MA, USA, http://www.thermofisher.com) and RNA quality was assessed using the Agilent 2100 bioanalyzer (Agilent Technologies, Santa Clara, CA, USA, http://www.alliedelec.com/). cDNA libraries were prepared using the Illumina TruSeq Sample Prep kit (Illumina Inc. San Diego, CA, USA, http://www.illumina.com) according to the manufacturers protocol. Library fragments were barcoded and multiplexed for sequencing to obtain 100 nt single-end reads. Library preparation and sequencing was performed at the Roy J. Carver Biotechnology Center using the Illumina Genome HiSeq 2000 (Illumina Inc. San Diego, CA, USA, http://www.illumina.com) and Cassava pipeline 1.8. FASTQ files from all sequencing runs are located on the Small Read Archive (http://www.ncbi.nlm.nih.gov/sra), SRA089043, BioProject number PRJNA207354.

### Transcriptome analyses

Sequencing adapters were removed from the raw FASTQ files using Cutadapt (ver. 1.8) [73]. A quality cutoff of 20 was used to trim low-quality bases. Only reads with a minimum length of 36 nt after trimming were retained. Trimmed RNASeq reads were aligned to the soybean reference genome (ver 1.1) using TopHat (ver. 1.4.1) [74]. A minimum intron length of 5 and a maximum intron length of 60,000 bp was used. Fragments Per Kilobase of Exon Model per Million mapped read (FPKM) values were determined using Cufflinks (ver. 1.3.0) [75]. Differentially expressed genes were determined using edgeR [76] from count data generated from HTSeq [77]. Due to underlying heterogeneity among all plots across the entire field experiment, genes were considered significantly differentially expressed when they had an FDR-adjusted *p*-value less than 0.1. The soybean genome (ver 1.1) functional annotation was used for all gene annotations (https://phytozome.jgi.doe.gov/pz/portal.html). Gene ontology (GO) enrichment was performed using single enrichment analysis from Agrigo (http://bioinfo.cau.edu.cn/agriGO/) with *Glycine max* (ver. 1.1) reference.

### Multiple sequence alignment

Multiple sequence alignment was performed using Clustal Omega (http://www.ebi.ac.uk/Tools/msa/clustalo/) [78]. Peptide sequences for the analysis were downloaded from the following public databases and can be found in Additional file 8: Table S6.

## Additional files

Additional file 1: Table S1. Mapping statistics for all libraries used in the RNA-Seq analysis. Reads were trimmed with Cutadapt v1.8 [73] and aligned to the soybean reference genome v1.1 with TopHat v.1.4.1 [74] (XLSX 31 kb)

Additional file 2: Table S2. Significantly differentially expressed genes in the soybean seed coat to elevated temperature. Genes were considered differentially expressed with a FDR-adjusted *p*-value of <0.10. logFC = log fold-change; logCPM = log counts per million; FDR = false discovery rate (XLSX 141 kb)

Additional file 3: Table S3. Fragment per Kilobase per Million Mapped (FPKM) reads for all abiotic stress treatments. FPKM values were generated using Cufflinks v1.3.0 [75] (XLSX 13221 kb)

Additional file 4: Figure S1. GO enrichment of genes with significantly increased expression in elevated temperature compared to control. Figure generated with Agrigo (http://bioinfo.cau.edu.cn/agriGO/) (PNG 410 kb)

Additional file 5: Figure. S2. Multiple sequence alignment output for MCM genes in soybean, Arabidopsis and maize. Analysis output from Clustal Omega [78] (PDF 451 kb)

Additional file 6: Table S4. Percent identity matrix for multiple sequence alignment of MCM peptide sequences. Values represent percent amino acid identity. Values generated using Clustal Omega [78] (XLSX 52 kb)

Additional file 7: Table S5. Expression values of soybean MCM genes from the soybean RNASeq expression atlas. Values generated from https://soybase.org (XLSX 48 kb)

Additional file 8: Table S6. Reference sequences used in multiple sequence alignment. Alignment completed with Clustal Omega [78] (XLSX 50 kb)

## References

[1] Ciais P, Reichstein M, Viovy N, Granier A, Ogee J, Allard V (2005). Europe-wide reduction in primary productivity caused by the heat and drought in 2003. Nature.

[2] Lobell DB, Field CB. Global scale climate - crop yield relationships and the impacts of recent warming. Environ Res Lett. 2007;2

[3] Van Dingenen R, Dentener FJ, Raes F, Krol MC, Emberson L, Cofala J (2009). (2009) the global impact of ozone on agricultural crop yields under current and future air quality legislation. Atmos. Environment.

[4] Fedoroff NV, Battisti DS, Beachy RN, Cooper PJM, Fischhoff DA, Hodges CN (2010). Radically rethinking agriculture for the 21st century. Science.

[5] Teixeira EI, Fischer G, van Velthuizen H, Walter C, Ewert F (2013). Global hot-spots of heat stress on agricultural crops due to climate change. Agric For Meteorol.

[6] IPCC (2013). Summary for policymakers. In: climate change 2013: the physical science basis. Contribution of working group I to the fifth assessment report of the intergovernmental panel on climate change Cambridge.

[7] Dai AG (2011). Drought under global warming: a review. Wires. Clim Chang.

[8] Rummukainen M (2012). Changes in climate and weather extremes in the 21st century. Wires. Clim Chang.

[9] Ainsworth EA, Yendrek CR, Sitch S, Collins WJ, Emberson LD (2012). The effects of tropospheric ozone on net primary productivity and implications for climate change. Annu Rev Plant Biol.

[10] Tuberosa R, Salvi S (2006). Genomics-based approaches to improve drought tolerance of crops. Trends Plant Sci.

[11] Ahuja I, de Vos RCH, Bones AM, Hall RD (2010). Plant molecular stress responses face climate change. Trends Plant Sci.

[12] Le DT, Nishiyama R, Watanabe Y, Tanaka M, Seki M, Ham LH, Yamaguchi-Shinozaki K, Shinozaki K, Tran LSP. Differential gene expression in soybean leaf tissues at late developmental stages under drought stress revealed by genome-wide transcriptome analysis. PLoS One. 2012;7 10.1371/journal.pone.0049522 PMC3505142 23189148

[13] Naika M, Shameer K, Mathew OK, Gowda R, Sowdhamini R. STIFDB2: An updated version of plant stress-responsive transcription factor database with additional stress signals, stress-responsive transcription factor binding sites and stress-responsive genes in Arabidopsis and rice. Plant Cell Physiol. 2013;54:e8(1–15). 10.1093/pcp/pcs185 PMC3583027 23314754

[14] Zhang H, Sonnewald U (2017). Differences and commonalities of plant responses to single and combined stresses. Plant J.

[15] Mittler R (2006). Abiotic stress, the field environment and stress combination. Trends Plant Sci.

[16] Leakey ADB, Ainsworth EA, Bernard SM, Markelz RJC, Ort DR, Placella SA, Rogers A, Smith MD, Sudderth EA, Weston DJ, Wullschleger SD, Yuan SH (2009). Gene expression profiling: opening the black box of plant ecosystem responses to global change. Glob Chang Biol.

[17] Hirayama T, Shinozaki K (2010). Research on plant abiotic stress responses in the post-genome era: past, present and future. Plant J.

[18] Roy SJ, Tucker EJ, Tester M (2011). Genetic analysis of abiotic stress tolerance in crops. Curr Opin Plant Biol.

[19] Sinclair TR (2011). Challenges in breeding for yield increase for drought. Trends Plant Sci.

[20] Richards CL, Rosas U, Banta J, Bhambhra N, Purugganan MD (2012). Genome-wide patterns of Arabidopsis gene expression in nature. PLoS Genet.

[21] Ruiz-Vera UM, Siebers M, Gray SB, Drag DW, Rosenthal DM, Kimball BA, Ort DR, Bernacchi CJ (2013). Global warming can negate the expected CO_2_ stimulation in photosynthesis and productivity for soybean grown in the midwestern United States. Plant Physiol.

[22] Siebers MH, Yendrek GR, Drag D, Locke AM, Acosta LR, Leakey ADB, Ainsworth AE, Cj B, Ort DR (2015). Heat waves imposed during early pod development in soybean (*Glyxine max*) cause significant yield loss despite a rapid recovery from oxidative stress. Global Chane. Biol.

[23] Gray SB, Dermody O, Klein SP, Locke AM, McGrath JM, Paul RE, Rosenthal DM, Ruiz-Vera UM, Siebers MH, Strellneer R, Ainsworth EA, Bernacchi CJ, Long SP, Ort DR, Leakey ADB. Intensifying drought eliminates the expected benefits of elevated carbon dioxide for soybean. Nature Plants. 2016; 10.1038/NPLANTS.2016.132. 10.1038/nplants.2016.132 27595230

[24] Schafleitner R, Rosales ROG, Gaudin A, Aliaga CAA, Martinez GN, Marca LRT, Bolivar LA, Delgado FM, Simon R, Bonierbale M (2007). Capturing candidate drought tolerance traits in two native Andean potato clones by transcription profiling of field grown plants under water stress. Plant Physiol Bioch.

[25] Leakey ADB, Xu F, Gillespie KM, McGrath JM, Ainsworth EA, Ort DR (2009). Genomic basis for stimulated respiration by plants growing under elevated carbon dioxide. Proc of the Natl Acad Sci, USA.

[26] Gillespie KM, Xu FX, Richter KT, McGrath JM, Markelz RJC, Ort DR, Leakey ADB, Ainsworth EA (2012). Greater antioxidant and respiratory metabolism in field-grown soybean exposed to elevated O_3_ under both ambient and elevated CO_2_. Plant Cell and Environ.

[27] Ainsworth EA, Yendrek CR, Skoneczka JA, Long SP (2012). Accelerating yield potential in soybean: potential targets for biotechnological improvement. Plant Cell Environ.

[28] Le BH, Wagmaister JA, Kawashima T, Bui AQ, Harada JJ, Goldberg RB (2007). Using genomics to study legume seed development. Plant Physiol.

[29] Jones SI, Gonzalez DO, Vodkin LO. Flux of transcript patterns during soybean seed development. BMC Genomics. 2010;11 10.1186/1471-2164-11-136 PMC2846912 20181280

[30] Weber H, Borisjuk L, Wobus U (2005). Molecular physiology of legume seed development. Annu Rev Plant Biol.

[31] Ranathunge K, Shao SQ, Qutob D, Gijzen M, Peterson CA, Bernards MA (2010). Properties of the seed coat cuticle change during development. Planta.

[32] Sreenivasulu N, Wobus U (2013). Seed development programs: a systems biology-based comparison between dicots and monocots. Annu Rev Plant Biol.

[33] Qutob D, Ma F, Peterson CA, Bernards MA, Gijzen M (2008). Structural and permeability properties off the soybean seed coat. Botany.

[34] Patrick JW, Offler CE (2001). Compartmentation of transport and transfer events in developing seeds. J Exp Bot.

[35] Weber H, Borisjuk L, Heim U, Buchner P, Wobus U (1995). Seed coat-associated invertases of fava bean control both unloading and storage functions – cloning of cDNAs and cell type-specific expression. Plant Cell.

[36] Weber H, Borisjuk L, Wobus U (1996). Controlling seed development and seed size in *Viciafaba*: a role for seed coat-associated invertases and carbohydrate state. Plant J.

[37] Weber H, Buchner P, Borisjuk L, Wobus U (1996). Sucrose metabolism during cotyledon development of *Viciafaba* L is controlled by the concerted action of both sucrose-phosphate synthase and sucrose synthase: expression patterns, metabolic regulation and implications for seed development. Plant J.

[38] Dornbos DL, Mullen RE (1991). Influence of stress during soybean seed fill on seed weight, germination, and seedling growth-rate. Can J Plant Sci.

[39] Egli DB, TeKrony DM, Heitholt JJ, Rupe J (2005). Air temperature during seed filling and soybean seed germination and vigor. Crop Sci.

[40] Smith JR, Mengistu A, Nelson RL, Paris RL (2008). Identification of soybean accessions with high germinability in high temperature environments. Crop Sci.

[41] Gallardo K, Firnhaber C, Zuber H, Hericher D, Belghazi M, Henry C, Kuster H, Thompson RA (2007). Combined proteome and transcriptome analysis of developing *Medicagotruncatula* seeds. Mol Cell Proteomics.

[42] Jones SI, Vodkin LO. Using RNA-Seq to profile soybean seed development from fertilization to maturity. PLoS One. 2013;8 10.1371/journal.pone.0059270 PMC3598657 23555009

[43] Verdier J, Dessaint F, Schneider C, Abirached-Darmency MA (2013). Combined histology and transcriptome analysis unravels novel questions on *Medicagotruncatula* seed coat. J Exp Bot.

[44] Kovinich N, Saleem A, Arnason JT, Miki B. Combined analysis of transcriptome and metabolite data reveals extensive differences between black and brown nearly-isogenic soybean (*Glycine max*) seed coats enabling the identification of pigment isogenes. BMC Genomics. 2011;12 10.1186/1471-2164-12-381 PMC3163566 21801362

[45] Hirsch CN, Foerster JM, Johnson JM, Sekhon RS, Muttoni G, Vaillancourt B, Peñagaricano F, Lindquist E, Pedraza MA, Barry K, de Leon N, Kaeppler SM, Buell CR (2014). Insights into the maize pan-genome and pan-transcriptome. Plant Cell.

[46] Dang HQ, Tran NQ, Gill SS, Tuteja R, Tuteja NA (2011). Single subunit MCM6 from pea promotes salinity stress tolerance without affecting yield. Plant Mol Biol.

[47] Tuteja N, Tran NQ, Dang HQ, Tuteja R, Plant MCM (2011). Proteins: role in DNA replication and beyond. Plant Mol Biol.

[48] Wanner LA, Junttila O (1999). Cold-induced freezing tolerance in Arabidopsis. Plant Physiol.

[49] Kaur S, Gupta AK, Kaur N (2000). Effect of GA_3_, kinetin and indole acetic acid on carbohydrate metabolism in chickpea seedlings germinating under water stress. Plant Growth Regul.

[50] Gupta AK, Kaur N (2005). Sugar signalling and gene expression in relation to carbohydrate metabolism under abiotic stresses in plants. J Biosci.

[51] Betzelberger AM, Yendrek CR, Sun JD, Leisner CP, Nelson RL, Ort DR, Ainsworth EA (2012). Ozone exposure response for U.S. soybean cultivars: linear reductions in photosynthetic potential, biomass, and yield. Plant Physiol.

[52] Mittler R (2006). Abiotic stress, the field environment and stress combination. Trends Plant Sci.

[53] Bell SP, Dutta ADNA (2002). Replication in eukaryotic cells. Annu Rev Biochem.

[54] Shultz RW, Lee TJ, Allen GC, Thompson WF, Hanley-Bowdoin L (2009). Dynamic localization of the DNA replication proteins MCM5 and MCM7 in plants. Plant Physiol.

[55] Lee TJ, Pascuzzi PE, Settlage SB, Shultz RW, Tanurdzic M, Rabinowicz PD, Menges M, Zheng P, Main D, Murray JA, Sosinski B, Allen GC, Martienssen RA, Hanley-Bowdoin L, Vaugh MW, Thompson WF (2010). Arabidopsis Thaliana chromosome 4 replicates in two phases that correlate with chromatin state. PLoS Genet.

[56] Costas C, de la Paz Sanchez M, Stroud H, Yu Y, Oliveros JC, Feng S, Benguria A, Lopez-Vidriero I, Zhang X, Solano R, Jacobsen SE, Guitierrez C (2011). Genome-wide mapping of Arabidopsis Thaliana origins of DNA replication and their associated epigenetic marks. Nat Struct Mol Biol.

[57] Springer PS, McCombie WR, Sundaresan V, Martienssen RA (1995). Gene trap tagging of *PROLIFERA*, an essential *MCM2-3-5* like gene in *Arabidopsis*. Science.

[58] Springer PS, Holding DR, Groover A, Yordan C, Martienssen RA (2000). The essential Mcm7 protein PROLIFERA is localized in the nucleus of dividing cells during the G_1_ phase and is required maternally for early *Arabidopsis* development. Development.

[59] Sabelli PA, Burgess SR, Kush AK, Young MR, Shewry PR (1996). cDNA cloing and characterization of a maize homologue of the MCM proteins required for the initiation of DNA replication. Mol Gen Genet.

[60] Bastida M, Puigdomenech P (2002). Specific expression of *ZmPRL*, the maize homolog of MCM7, during early embryogenesis. Plant Sci.

[61] Holding DR, Springer PS (2002). The Arabidopsis gene *PROLIFERA* is required for proper cytokinesis during seed development. Planta.

[62] Stevens R, Mariconti L (2002). Rossignol p, Perennes C, Cella R, Bergounioux C. Two E2F sites in the Arabidopsis *MCM3* promoter have different roles in cell cycle activation and meristem expression. J Biol Chem.

[63] Dresselhaus T, Srilunchang KO, Leljak-Levanic D, Schreiber DN, Garg P (2006). The fertilization-induced DNA replication factor MCM6 of maize shuttles between cytoplasm and nucleus, and is essential for plant growth and development. Plant Physiol.

[64] Ni DA, Sozzani R, Blanchet S, Domenichini S, Reuzeau C, Cella R, Bergounioux C, Raynaud C (2009). The Arabidopsis MCM2 gene is essential to embryo development and its over-expression alters root meristem function. New Phytol.

[65] Shanmugam A, Robin AHK, Thamilarasan SK, Vijayakumar H, Natarajan S, Kim H-T, Park J-I, Nou I-S (2017). Genome-wide characterization and stress-responsive expression profiling of *MCM* genes in *Brassica oleracea* and *Brassica rapa*. J Plant Biol.

[66] Morgan PB, Bernacchi CJ, Ort DR, Long SP (2004). An in vivo analysis of the effect of season-long open-air elevation of ozone to anticipated 2050 levels on photosynthesis in soybean. Plant Physiol.

[67] Gray SB, Strellner RS, Puthuval KK, Ng C, Shulman RE, Siebers MH, Rogers A, Leakey ADB (2013). Minirhizotron imaging reveals that nodulation of field-grown soybean is enhanced by free-air CO_2_ enrichment only when combined with drought stress. Funct Plant Biol.

[68] Jones MGK, Outlaw WH, Lowry OH (1977). Enzymic assay of 10^−7^to 10^−14^moles of sucrose in plant tissues. Plant Physiol.

[69] Hendriks JHM, Kolbe A, Gibon Y, Stitt M, Geigenberger P (2003). ADP-glucose pyrophosphorylase is activated by posttranslational redox-modification in response to light and to sugars in leaves of Arabidopsis and other plant species. Plant Physiol.

[70] Mudge JF, Baker LF, Edge CB, Houlahan JE. Setting an optimal alpha yhat minimizes errors in null hypothesis significance tests. PLoS One. 2012;7 10.1371/journal.pone.0032734 PMC3289673 22389720

[71] Cohen JE (1988). Statistical power analysis for the behavioral sciences.

[72] Locke AM, Sack L, Bernacchi CJ, Ort DR (2013). Soybean leaf hydraulic conductance does not acclimate to growth at elevated [CO_2_] or temperature in growth chambers or in the field. Ann Bot-London.

[73] Martin M (2011). Cutadapt removes adapter sequences from high-throughput sequencing reads. EMBNet. Journal.

[74] Trapnell C, Pachter L, Salzber SL (2009). TopHat: discovering splice junctions with RNA-Seq. Bioinformatics.

[75] Trapnell C, Williams BA, Pertea G, Mortazavi A, Kwan G, van Baren MJ, Salzber SL, Wold BJ, Pachter L (2010). Transcript assembly and abundance estimation from RNA-Seq reveals thousands of new transcripts and switching among isoforms. Nat Biotechnol.

[76] Robinson MD, McCarthy DJ, Smyth GK (2010). edgeR: a Bioconductor package for differential expression analysis of digital gene expression data. Bioinformatics.

[77] Anders S, Pyl PT (2015). Huber W HTSeq—a Python framework to work with high-throughput.sequencing data. Bioinformatics.

[78] Sievers F, Wilm A, Dineen DG, Gibson TJ, Karplus K, Li W, Lopez R, McWilliam H, Remmert M, Söding J, Thompson JD, Higgins D (2011). Fast, scalable generation of high-quality protein multiple sequence alignments using Clustal omega. Mol Syst Biol.

