# Supplementary figures and images for: Physiological and transcriptomic responses in the seed coat of field-grown soybean (Glycine max L. Merr.) to abiotic stress
Source: BMC Plant Biol. 2017 Dec 12;17:242. doi: 10.1186/s12870-017-1188-y (PMC5727933; doi:10.1186/s12870-017-1188-y)

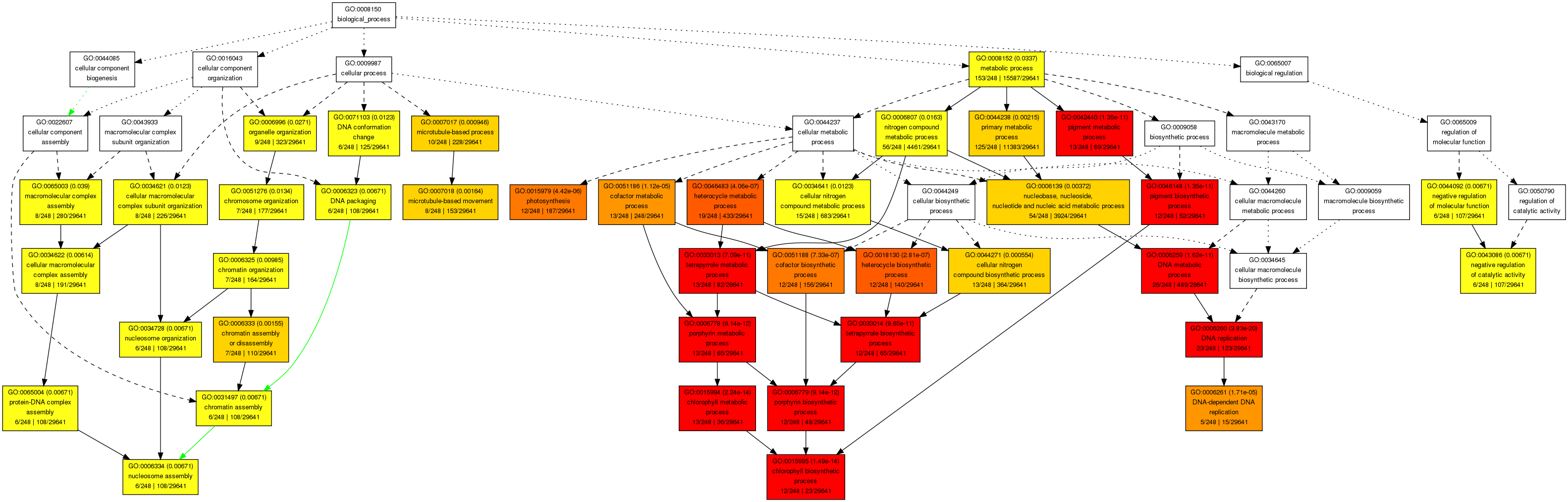

Supplement: Supplementary file 4 — GO enrichment of genes with significantly increased expression in elevated temperature compared to control. Figure generated with Agrigo (http://bioinfo.cau.edu.cn/agriGO/) (PNG 410 kb) [file 12870_2017_1188_MOESM4_ESM.png]
