# Supplementary material for: Physiological and transcriptomic responses in the seed coat of field-grown soybean (Glycine max L. Merr.) to abiotic stress
Source: BMC Plant Biol. 2017 Dec 12;17:242. doi: 10.1186/s12870-017-1188-y (PMC5727933; doi:10.1186/s12870-017-1188-y)
Supplement: Supplementary file 5 — Multiple sequence alignment output for MCM genes in soybean, Arabidopsis and maize. Analysis output from Clustal Omega [78] (PDF 451 kb) [file 12870_2017_1188_MOESM5_ESM.pdf]

CLUSTAL O(1.2.4) multiple sequence alignment

```
BoMCM6_2                ------------------------------------------------------------    0
BoMCM6_1                ------------------------------------------------------------    0
AtMCM6                  ------------------------------------------------------------    0
BrMCM6                  ------------------------------------------------------------    0
ZmMCM6                  ------------------------------------------------------------    0
PsMCM6                  ------------------------------------------------------------    0
MCM6_Glyma09g05240      ------------------------------------------------------------    0
MCM6_Glyma15g16570      ------------------------------------------------------------    0
AtMCM2                  MAGENS----DNEPSSPASPSSAGF-NTDQLPISTSQNS------ENFSDEEEAAVDTQV    49
BoMCM2_1                MAGEDS----DNQPSS---PSSAGF-NTDQLPFCTSQNS------ENFSDG-EAEVDPQV    45
BrMCM2_1                MAGEDS----DNQPSSPASPSSAGF-NTDQLPFSTSQNS------ENFSDEEEAEVDPQV    49
BoMCM2_2                MAGEDS----DNQPSSPASPSSAGF-NTDQLPFSTSQNS------ENISDE-EAEVDPQI    48
BrMCM2_2                MAGEDS----ENQPSSPASPSSAGF-NTDQLPFSTSQNS------ENFSDE-EAEVDPQI    48
ZmMCM2                  MDD--S---ENNAPS---TPGSPGF-STDRLPPNTTTSRGATDPSSYSDDDGEAEVDPHV    51
PsMCM2                  MEPGIP----PSTPDSP-TSPSIGF-NTDQLPHTHTSR---------TSEDDEASVDPDI    45
MCM2_Glyma07g36680      ----------MASDPESP-TSPSVGF-NTDQLPHTHTSR---------ASEDDEASVDPDI    40
MCM2_Glyma17g03920      MAPENP---STPDPESP-TSPSVGF-NTDQLPHTHTSH---------ASQDDEASVDPDI    46
ZmMCM5                  ------------------------------------------------------------    0
AtMCM5                  ------------------------------------------------------------    0
BoMCM5                  ------------------------------------------------------------    0
BrMCM5                  ------------------------------------------------------------    0
PsMCM5                  ------------------------------------------------------------    0
MCM5_Glyma13g22420      ------------------------------------------------------------    0
MCM5_Glyma17g11220      ------------------------------------------------------------    0
ZmMCM3                  ------------------------------------------------------------    0
AtMCM3                  ------------------------------------------------------------    0
BrMCM3_1                ------------------------------------------------------------    0
BoMCM3                  ------------------------------------------------------------    0
BrMCM3_2                ------------------------------------------------------------    0
MCM3_Glyma05g25980      ------------------------------------------------------------    0
MCM3_Glyma08g08920      ------------------------------------------------------------    0
PsMCM3                  ------------------------------------------------------------    0
AtMCM7                  ------------------------------------------------------------    0
BoMCM7_2                ------------------------------------------------------------    0
BrMCM7_1                ------------------------------------------------------------    0
BoMCM7_1                ------------------------------------------------------------    0
BrMCM7_2                ------------------------------------------------------------    0
ZmMCM7                  ------------------------------------------------------------    0
PsMCM7                  ------------------------------------------------------------    0
MCM7_Glyma03g37770      ------------------------------------------------------------    0
MCM7_Glyma19g40370      ------------------------------------------------------------    0
ZmMCM4                  MASDGGGS-SPPPASSPYGRPSSPLAVTNSSPSQPT------------------------    35
AtMCM4                  MASDSSLGNTNDGPPSPGENVSSPIENTYSSPAALH------------------------    36
BoMCM4                  MASDSPPANTNDGPSSPGENASSPIGNTYSSPSSLR------------------------    36
BrMCM4                  MASDSPPANTNDGPSSPGENASSPIGNTYSSPSSLR------------------------    36
MCM4_Glyma11g12110      MASDSSPPNFNNGPSSPDDSLSSPIGNTFSSPAS--------------------------    34
MCM4_Glyma12g04320      MASDSSPLNFNNGPSSPDDSLSSPIGNTFSSPAS--------------------------    34
PsMCM4                  MASNSSPMDF--DPSSPDIPPSSPIPNTLSTPGR--------------------------    32


BoMCM6_2                ------------------------------------------------------------    0
BoMCM6_1                ------------------------------------------------------------    0
AtMCM6                  ------------------------------------------------------------    0
BrMCM6                  ------------------------------------------------------------    0
ZmMCM6                  ------------------------------------------------------------    0
PsMCM6                  ------------------------------------------------------------    0
MCM6_Glyma09g05240      ------------------------------------------------------------    0
```

```
MCM6_Glyma15g16570      ------------------------------------------------------------    0
AtMCM2                  IRDEPDE---AEDEEEEEGEDLFNDTFMNDYRKMDENDQYESNGIDDSVDDERDLGQAML    106
BoMCM2_1                IRDEPED---EE--EEEEGEDLFNDNFHQDYEKRDDQDQYESVGLDDSVVDDRGLGQIAL    100
BrMCM2_1                IRDEPED---EEEEEEEEGEDLFNDNFHQDYEKRDEQDQYESVGLDDSVVDNRGLGQIAL    106
BoMCM2_2                IRDEPEE---E---EEEDGEDLFNDNFLQDYGRRDEQDQYESVGLDDSVDDDRDLGQIAL    102
BrMCM2_2                IRDEPEE---PEE-EEEDGEDLFNDNFLQDYGKRDEQDQYESVGLDDSVDDDRDLGQIAL    104
ZmMCM2                  LPDDDDPAAVAAPDEDEEGEDLFNDNYLDDYRRMDEHDQYESVGLDDSLEDERNLDEIMA    111
PsMCM2                  IRDEPE------PEEDEDGEDLYNDNFLEDYRRMDEADQFESVGLDDSVEDERDFDQIME    99
MCM2_Glyma07g36680      VRDEIE---EQPEE-EEDGEDLYNDNFLDDYKRMDEADQFESFGLDDSLEDDRDFDQIMQ    96
MCM2_Glyma17g03920      VRDDIE---EQPEEEEEDGEDLYNDNFLDDYKRMDEADQFESFGLDDSLEDDRDFDQIMQ    103
ZmMCM5                  ------------------------------------------------------------    0
AtMCM5                  ------------------------------------------------------------    0
BoMCM5                  ------------------------------------------------------------    0
BrMCM5                  ------------------------------------------------------------    0
PsMCM5                  ------------------------------------------------------------    0
MCM5_Glyma13g22420      ------------------------------------------------------------    0
MCM5_Glyma17g11220      ------------------------------------------------------------    0
ZmMCM3                  ------------------------------------------------------------    0
AtMCM3                  ------------------------------------------------------------    0
BrMCM3_1                ------------------------------------------------------------    0
BoMCM3                  ------------------------------------------------------------    0
BrMCM3_2                ------------------------------------------------------------    0
MCM3_Glyma05g25980      ------------------------------------------------------------    0
MCM3_Glyma08g08920      ------------------------------------------------------------    0
PsMCM3                  ------------------------------------------------------------    0
AtMCM7                  ------------------------------------------------------------    0
BoMCM7_2                ------------------------------------------------------------    0
BrMCM7_1                ------------------------------------------------------------    0
BoMCM7_1                ------------------------------------------------------------    0
BrMCM7_2                ------------------------------------------------------------    0
ZmMCM7                  ------------------------------------------------------------    0
PsMCM7                  ------------------------------------------------------------    0
MCM7_Glyma03g37770      ------------------------------------------------------------    0
MCM7_Glyma19g40370      ------------------------------------------------------------    0
ZmMCM4                  ------------------------------------------------------------    35
AtMCM4                  ------------------------------------------------------------    36
BoMCM4                  ------------------------------------------------------------    36
BrMCM4                  ------------------------------------------------------------    36
MCM4_Glyma11g12110      ------------------------------------------------------------    34
MCM4_Glyma12g04320      ------------------------------------------------------------    34
PsMCM4                  ------------------------------------------------------------    32


BoMCM6_2                ------------------------------------------------------------    0
BoMCM6_1                ------------------------------------------------------------    0
AtMCM6                  ------------------------------------------------------------    0
BrMCM6                  ------------------------------------------------------------    0
ZmMCM6                  ------------------------------------------------------------    0
PsMCM6                  ------------------------------------------------------------    0
MCM6_Glyma09g05240      ------------------------------------------------------------    0
MCM6_Glyma15g16570      ------------------------------------------------------------    0
AtMCM2                  DRRAADADLDARENRL---ANRKLPHLLHDN-DSDDWNYRPSKRSRTTVPPRG------N    156
BoMCM2_1                DRRAAEAVLDARESRL---ANRKLPHLLHDN-DSDDSNYRPSKRARAAVPPRG------N    150
BrMCM2_1                DRRAAEAVLDARETRL---ANRKLPHLLHDN-DSDDSNYRPSKRARAAVPPRG------N    156
BoMCM2_2                DRRAADAVLDARESRF---ANRKLPHLLHDNNDSDDWNYRPSKRARAAVPPRD------P    153
BrMCM2_2                DRRAADAVLDARESRF---ANRKLPHLLHDN-DSDEWNYRPSKRARAAVPPRG------S    154
ZmMCM2                  DRRAAEAELDAREVRTGATADRKLPRMLHDQDTDDDTNFRRPKRHRASFRQPSGPRTPRS    171
PsMCM2                  DRRAAEVELDTRDGRA--SNRTKLPQLLHDQDTDDDS-YRPSKRARADHRS----SVPPS    152
MCM2_Glyma07g36680      DRRAAEIELEARDGRA--SNRNKLPQLLHDQDTDDDN-HRSSKRARADFRP----SA-AS    148
MCM2_Glyma17g03920      DRRAAEVELEARDGRA--SNRNKLPQLLHDQDTDDDN-HRSSKRARADFRP----SV-AS    155
ZmMCM5                  ------------------------------------------------------------    0
```

```
BoMCM5                 ------------------------------------------------------------    0
BrMCM5                 ------------------------------------------------------------    0
PsMCM5                 ------------------------------------------------------------    0
MCM5_Glyma13g22420     ------------------------------------------------------------    0
MCM5_Glyma17g11220     ------------------------------------------------------------    0
ZmMCM3                 ------------------------------------------------------------    0
AtMCM3                 ------------------------------------------------------------    0
BrMCM3_1               ------------------------------------------------------------    0
BoMCM3                 ------------------------------------------------------------    0
BrMCM3_2               ------------------------------------------------------------    0
MCM3_Glyma05g25980     ------------------------------------------------------------    0
MCM3_Glyma08g08920     ------------------------------------------------------------    0
PsMCM3                 ------------------------------------------------------------    0
AtMCM7                 ------------------------------------------------------------    0
BoMCM7_2               ------------------------------------------------------------    0
BrMCM7_1               ------------------------------------------------------------    0
BoMCM7_1               ------------------------------------------------------------    0
BrMCM7_2               ------------------------------------------------------------    0
ZmMCM7                 ------------------------------------------------------------    0
PsMCM7                 ------------------------------------------------------------    0
MCM7_Glyma03g37770     ------------------------------------------------------------    0
MCM7_Glyma19g40370     ------------------------------------------------------------    0
ZmMCM4                 RRSG------GRRRRGSASPYASSPSLG---------GFE--------TPPHPGRRTPS-    71
AtMCM4                 RRRR----------GRSSTPT----------------QFA--------TPPPPPSRLASS    62
BoMCM4                 SRRR----------ARSSTPS----------------QFA--------TPPP-PSRLGVP    61
BrMCM4                 TRRR----------ARSSTPS----------------QFA--------TPPP-PSRLGVP    61
MCM4_Glyma11g12110     -RRR----------RRSSTPS----------------AFA--------TPSERRSRFASS    59
MCM4_Glyma12g04320     -RRR----------RRSSTPS----------------AFA--------TPSERRSRFASS    59
PsMCM4                 -RRR----------RQSTTPS----------------AYG--------TPQPNRSRLASS    57


BoMCM6_2               ------------------------------------------------------------    0
BoMCM6_1               ------------------------------------------------------------    0
AtMCM6                 ------------------------------------------------------------    0
BrMCM6                 ------------------------------------------------------------    0
ZmMCM6                 ------------------------------------------------------------    0
PsMCM6                 ------------------------------------------------------------    0
MCM6_Glyma09g05240     ------------------------------------------------------------    0
MCM6_Glyma15g16570     ------------------------------------------------------------    0
AtMCM2                 GGDPDGNPPS----SPGVS--------------------QPDISMTDQTDDYQDEDDNDD    192
BoMCM2_1               GGDPDGNP-S----SPGTS--------------------LPDVSMTDQTDDYQDED---D    182
BrMCM2_1               GRDPDGNP-S----SP-----------------------LPDVSMTDQTDDYQDED---D    185
BoMCM2_2               DGDPS-------------S--------------------QPDVSMTDQTDDYQDEDDNDD    180
BrMCM2_2               GGDPDGNP-S----SPGTS--------------------QPDVSMTDQTDDYQDEDDNDD    189
ZmMCM2                 DDDGDGATPS----SPGRSHRG--------------MSSGGDVPMTDQTDDDPYEDEFDE    213
PsMCM2                 EDDLDG-MNS----SPGRSQRG--------------QHSRDDNPTTDQNEDDQYEDDFDD    193
MCM2_Glyma07g36680     DDDLDG-MQS----SPGRSQRG--------------HP-REDVLMTDQTEDDRDDDDFD-    187
MCM2_Glyma17g03920     DDDFDG-MQS----SPGRSQR---------------HS-REDILMTDQTEDDRDDDDFD-    193
ZmMCM5                 --------------------------MSG-WDEGA-VFYSDQAQFPRG----G-------    21
AtMCM5                 --------------------------MSG-WDEGA-VYYSDQPQFP--------------    18
BoMCM5                 --------------------------MSG-WDEGA-VYYSDQPQFP--------------    18
BrMCM5                 --------------------------MSG-WDEGA-VYYSDQPQFP--------------    18
PsMCM5                 --------------------------MSG-WDEGG-VYYSDQAHSWDD----G-------    21
MCM5_Glyma13g22420     --------------------------MSG-WDEGA-VYYSDQALAGDD----G-------    21
MCM5_Glyma17g11220     --------------------------MSG-WDEGA-VYYSDQALAGDD----G-------    21
ZmMCM3                 ------------------------------------------------------------    0
AtMCM3                 ------------------------------------------------------------    0
BrMCM3_1               ------------------------------------------------------------    0
BoMCM3                 ------------------------------------------------------------    0
BrMCM3_2               ------------------------------------------------------------    0
```

```
MCM3_Glyma05g25980    ------------------------------------------------------------    0
MCM3_Glyma08g08920    ------------------------------------------------------------    0
PsMCM3                ------------------------------------------------------------    0
AtMCM7                ------------------------------------------------------------    0
BoMCM7_2              ------------------------------------------------------------    0
BrMCM7_1              ------------------------------------------------------------    0
BoMCM7_1              ------------------------------------------------------------    0
BrMCM7_2              ------------------------------------------------------------    0
ZmMCM7                ------------------------------------------------------------    0
PsMCM7                ------------------------------------------------------------    0
MCM7_Glyma03g37770    ------------------------------------------------------------    0
MCM7_Glyma19g40370    ------------------------------------------------------------    0
ZmMCM4                GA-GAGAPR----------QPRQNSTGRFPPTPSTPMSTDDVPPSSEAGDDET-------    113
AtMCM4                NS-TPPTSRPSAARSKGRNGHGGGGGGGGGGDPGTPMSTDEPLPSSDDG-----------    110
BoMCM4                SS-TPPTSRPSAARSN---------------RPATPSHTDEPPPSSDDG-----------    94
BrMCM4                NS-TPPASRPSAARSN---------------RPATPSHTDEPPPSSDDG-----------    94
MCM4_Glyma11g12110    DA-TPTAPRS---------RQRS----GGGRVPATSTSTTDDVPASSDG---G-------    95
MCM4_Glyma12g04320    DA-TPTAPRS---------RQRGGSGGGGGHVPATPTSTTDDVPVSSDG---G-------    99
PsMCM4                DA-TPTPSRH---------RR-GGGIPSGRRVPATPTSTSDDIPMSSEG---G-------    96


BoMCM6_2              ------------------------------------------------------------    0
BoMCM6_1              MEAFGGFV---------MDEQA-IQVENVFLEFLKSFRLDA------------NKPGLYY    38
AtMCM6                MEAFGGFV---------MDEQA-IQVENVFLEFLKSFRLDA------------NKPELYY    38
BrMCM6                MEAFGGFV---------MDEQA-IQVENVFLEFLKSFRLDA------------NKPELYY    38
ZmMCM6                MEAFGGFF---------VDEKA-ARVENIFLEFLKRFKESD------------GAGEPFY    38
PsMCM6                MEAFGGYL---------VDEKA-VRVENAFLDFLKSFRS-G------------QRNELYY    37
MCM6_Glyma09g05240    MEAYGGFM---------IDEKA-VRVENAFLDFLKSFKSSS------------HRNELYY    38
MCM6_Glyma15g16570    MEAYGGFM---------VDEKA-VRVENAFLDFLKSFKSSS------------QRNELYY    38
AtMCM2                EAEFEMYRIQGTLREWVMRDEVRRFIAKKFKDFLLTYVKPK-----------NENGDIEY    241
BoMCM2_1              EAEFEMYRIQGSLREWVMRDEVRRFIAKKFRDFLLTYVKPN-----------SENG--EY    229
BrMCM2_1              EAEFEMYRIQGSLREWVMRDEVRRFIAKKFRDFLLTYVKPN-----------SENG--EY    232
BoMCM2_2              EAEFEMYRIQGSLREWVMRDEVRRFIAKKFRDFLLTFVKPN-----------NENG--EY    227
BrMCM2_2              EAEFEMYRIQGSLREWVMRDEVRRFIAKKFRDFLLTYVKPN-----------SENG--EY    236
ZmMCM2                EDEMNMYRVQGTLREWVTRDEVRRFIAKKFKEFLLTYVHPK-----------NDQGEFEY    262
PsMCM2                EAGYEMYRVQGTLREWVTRDEVRRFIARKFKDFLLTYVNPK-----------NEHGDFEY    242
MCM2_Glyma07g36680    -DGYEMYHVQGTLREWVTRDEVRRFIARKFKDFLLTYVNPK-----------NEHRDKEY    235
MCM2_Glyma17g03920    -DGYEMYHVQGTLREWVTRDEVRRFIARKFKDFLLTYLNPK-----------NEHRDKEY    241
ZmMCM5                -PGGDP-A---------ADLTR-HSALRKFKEFLRGFTGPT--------------GDFPY    55
AtMCM5                ---EAGDA---------ATISP-HAVLTKFKEFIRNFEIEQ--------------NCFPY    51
BoMCM5                ---EAGDA---------ATVSP-HAVMTKFKEFIRTFEIGQ--------------NCFPY    51
BrMCM5                ---EAGDA---------ATVSP-HAVMTKFKEFIRTFEIGQ--------------NCFPY    51
PsMCM5                -RGEA--E---------ATVSN-HTILQKFKEFIRNFETGN--------------NVFPY    54
MCM5_Glyma13g22420    -SGARGEA---------GTDSN-HSLIQKFKEFIRNFETTN--------------NVFPY    56
MCM5_Glyma17g11220    -AGARGEA---------GTDSN-HTLIQKFKEFIRNFETTN--------------NVFPY    56
ZmMCM3                -----------------MEINE--EAMAAHKRAFLDFLD-------------QDVGKGVY    28
AtMCM3                -----------------MDVPE--ETRLRHKRDFIQFLD------------------SMY    23
BrMCM3_1              -----------------MDVPE--ETRLRHKRDFIQFLE------------------NIY    23
BoMCM3                -----------------MDVPE--ETRLRHKRDFIQFLE------------------NIY    23
BrMCM3_2              -----------------MDVPE--ETRLRHKRDFIQFLE------------------NIY    23
MCM3_Glyma05g25980    -----------------MDLSE--EVRAAHKREFSDFLD-------------QDVGKGIY    28
MCM3_Glyma08g08920    -----------------MDLSE--EVRAAHKREFSDFLD-------------QDVGKGIY    28
PsMCM3                ------------------------------------------------------------    0
AtMCM7                ----------------MKDHDF-DGDKGLAKGFLENFADAN--------------GRSKY    29
BoMCM7_2              ----------------MKEHDF-EADKALAKEFLANFTDAS--------------GGSKY    29
BrMCM7_1              ----------------MKEHDF-EADKALAKEFLANFTYAS--------------GGSKY    29
BoMCM7_1              ----------------MKEHDF-EADKAVAKEFLANFTDAN--------------GRSKY    29
BrMCM7_2              ----------------MKEHDF-EADKAVAKEFLANFTDAN--------------GRSKY    29
ZmMCM7                ----------------MKNPDF-AADKALAKDFLSNFTGPH--------------GEPKY    29
PsMCM7                --------------MNTKGVDF-ASDKVLASRFLSEFADAN--------------GEAKY    31
MCM7_Glyma03g37770    --------------MSAKNLNF-DADTALAKDFLSNFADAN--------------GEAKY    31
```

```
MCM7_Glyma19g40370     --------------MSTKDLNF-DADTALAKDFLSNFADAN--------------GEAKY   31
ZmMCM4                 -DGGGGVD-ATPVFVWGTNISV-QDVNAAILRFLRHFRDPRDAGR-----VDPVMDEGKY   165
AtMCM4                 -EEDGGDD-TTPTFVWGTNISV-QDVKSAIEMFVKHFREARENS-------DDLFREGKY   160
BoMCM4                 -GEDGADD-ATPTFVWGTNISV-QDVKSAIEMFVKHFREAKESS-------DDLFREGKY   144
BrMCM4                 -GEDGADD-ATPTFVWGTNISV-QDVKSAIEMFVKHFREAKESS-------DDLFREGKY   144
MCM4_Glyma11g12110     -DGFDMDD-ARPTFVWGTNISV-EDVNDAIKRFLRNFRDASSSQGGDNDDGLHLHTEGKY   152
MCM4_Glyma12g04320     -DGFDMDD-ARPTFVWGTNISV-EDVNDAIKRFVRNFREASSSQGGDDDDGLHLHTEGKY   156
PsMCM4                 -DGYDMDD-AGPTYVWGTNISV-EDVNDAIQRFLKHFREQSTSQGDID----DLDTEGKY   149


BoMCM6_2               ------------------------------------------------------------   0
BoMCM6_1               EAEIEAIRGGES-TMMYIDFSHVMGFN------DALQKAIADEYLRFEPYLRNACKRFVI   91
AtMCM6                 EAEIEAIRGGES-TMMYIDFSHVMGFN------DALQKAIADEYLRFEPYLRNACKRFVI   91
BrMCM6                 EAEIEAIRGGES-TMMYIDFSHVMGFN------DALQRAIADEYLRFEPYVRNACKRFVI   91
ZmMCM6                 EAEMEVMRSRES-TTMYVDFAHVMRFN------DVLQKAISEEYLRFEPYLRNACKRFAL   91
PsMCM6                 EAEIEVMRANES-NTMFIDFEHVIRFS------DLLQKAISDEYLRFEPYLQNACKRFVM   90
MCM6_Glyma09g05240     EAEIELMKSNDS-NTMFVDFDHVIRFS------DLLQQTISDEYLRFEPYLKNACKRLVM   91
MCM6_Glyma15g16570     EAEIELMKSNES-NTMFIDFDHVIRFS------DLLQQTISDEYLRFEPYLKNACKRFVM   91
AtMCM2                 VRLINEMVSANK-CSLEIDYKEFIHVH-----PNIAIW-LADAPQPVLEVMEEVSEKVIF   294
BoMCM2_1               VRLINEIVSANK-CSLEIDYKEFIHVH-----PNIAIW-LADAPQPVLEVMEEVSEKVIF   282
BrMCM2_1               VRLINEMVSANK-CSLEIDYKEFIHVH-----PNIAIW-LADAPQPVLEVMEEVSEKVIF   285
BoMCM2_2               VRLINEMVSANK-CSLEIDYKEFIHVH-----PNIAIW-LADAPQPVLEVMEEVSEKVIF   280
BrMCM2_2               VRLINEMVSANK-CSLEIDYKEFIHVH-----PNIAIW-LADAPQPVLEVMEEVSEKVIF   289
ZmMCM2                 VRLINEMVLANK-CSLEIDYKQFIYIH-----PNIAIW-LADAPQSVLEVMEEVAKNVVF   315
PsMCM2                 VRLINEMVSANK-CSLEIDYKQFIYVH-----PNIAIW-LADAPHSVLEVMEDVAKSVVF   295
MCM2_Glyma07g36680     VWLINEMVSASK-CSLEIDYKQFIYVH-----PNIAIW-LADAPQSVLEVMEDVTKSVVF   288
MCM2_Glyma17g03920     VWLINEMVSASK-CSLEIDYKQFIYVH-----PNIAIW-LADAPQSVLEVMEDVTKNVVF   294
ZmMCM5                 R----ESLVHNR-DHVTVAIEDLDAFD-----AELSDK-IRKSPADYLPLFETAAAEVLA   104
AtMCM5                 R----EALLDNP-KRLVVHLEDLLSFD-----SDLPSL-IRSAPADYLPVFEKAAGEVLT   100
BoMCM5                 R----EALLDNP-KRLLVHLEDLLAFD-----SDLPSL-IRSAPADFLPVFEKAAGEVLA   100
BrMCM5                 R----EALLDNP-KRLLVHLEDLLSFD-----SDLPSL-IRSAPADFLPVFEKAAGEVLA   100
PsMCM5                 R----ESLLNNP-KFLVIDMEDLDSFD-----PDLPSK-LRSAPADILPLFETAAAQVLV   103
MCM5_Glyma13g22420     R----ESLLHNP-KFLLVDMGDLDTFD-----SDLPDK-LRSNPADVLPLFEAAAAQVLV   105
MCM5_Glyma17g11220     R----ESLIHNP-KFLLVDMGDLDTFD-----SELPAK-LRSNPADVLPLFETAAAQVLV   105
ZmMCM3                 MQAVRDMVQNKR-HRLIIGMDDLRNH-----NLDLARR-VIRTPGEYMQPASDAVSEVAR   81
AtMCM3                 MEEIKALVHQKR-HRLIINISDIHHHF---REV--ASR-ILKNPNEYMQSFCDAATEATR   76
BrMCM3_1               MDEIKSLVQNKR-HRLIVNVSDIHTHF---RESESSSR-ILKNPIEYMQSFCDAATEATR   78
BoMCM3                 MDEIKALVQNKR-HRLIVNVSDIHTHF---RESESSSR-ILKNPIEYMQSFCDAATEATR   78
BrMCM3_2               MDEIKALVQNKR-HRLIVNVSDIHTHF---RESESSSR-ILKNPIEYMQSFCDAATEATR   78
MCM3_Glyma05g25980     MDEIKTLINLKR-HRLIVNISDLHNF----R--DLGNR-ILRSPSEYMQPFCDAVTEATR   80
MCM3_Glyma08g08920     MDEIKTLINHKR-HRLIVNISDLHNF----R--DLGNR-ILRSPSEYMQPFCDAVTEATR   80
PsMCM3                 -----------------------------------------------MQSFCDAVTDAVR   13
AtMCM7                 MEILQEVSNRKI-RAIQVDLDDLFNYKD--ESEEFLGR-LTENTRRYVSIFSAAVDELLP   85
BoMCM7_2               LEILQEVANRKS-RAIQVDLQDLINYKGFDEFHEFMTR-LTENTRRYVSIFSSAIDELLP   87
BrMCM7_1               LEILQEVANRKT-RAIQVDLQDLINYKGFDEFHEFMTR-LTENTRRYVSIFSSAIDELLP   87
BoMCM7_1               LEILQEVANRKV-RAIQIDLQDLINDKGFDDFHEFIGR-LTENTRRYVSIFSSAIDELLP   87
BrMCM7_2               LEILQEVANRKV-RAIQIDLQDLINDKGFDDFHEFIGR-LTENTRRYVSIFSSAIDELLP   87
ZmMCM7                 LNILQDVANRKI-RAVQIELDDLFHYKDVD--EEFLQR-VTENTRRYIGIFAEAMDEIMP   85
PsMCM7                 INILQDVANHKT-RSVEIDLDDLMNYKDLD--EEFLTR-VTENTRRYVGIFADAIDELMP   87
MCM7_Glyma03g37770     MNILQDVANHKT-RAVQIDLEDLFNYKDLD--EEFLSR-VTDNTRRYIGIFSNAIDELMP   87
MCM7_Glyma19g40370     MNILQDVANHKT-RAVQIDLEDLFNYKDLD--EEFLSR-VTDNTRRYIGIFSNAIDELMP   87
ZmMCM4                 MRAIHRILELEGGESLDVDAHDVFDHD-----PDLYSK-MVRYPLEVLAIFDIV------   213
AtMCM4                 MVSIRKVIEIEG-EWIDVDAFDVFDYD-----PDLYNK-MVRYPLEVLAIFDIV------   207
BoMCM4                 MASIRKVIEIEG-EWIDVDAYDVFDYD-----PDLYNK-MVRYPLEVLAIFDIV------   191
BrMCM4                 MASIRKVIEIEG-EWIDVDAYDVFDYD-----PDLYNK-MVRYPLEVLAIFDIV------   191
MCM4_Glyma11g12110     EKLIRQVIEVEG-DSLDVDARDVFDHD-----PDLYTK-MVRYPLEVLAIFDLV------   199
MCM4_Glyma12g04320     EKLIRQVIDVEG-DSLDVDARDVFDHD-----PDLYTK-MVRYPLEVLAIFDLV------   203
PsMCM4                 EKLIKQVIELEG-ESIDVDARDVFDHD-----PDLYTK-MVRYPLEVLAIFDMV------   196


BoMCM6_2               -----------MNPE---------------------------------TPNKDINVSFYN   16
```

```
BoMCM6_1            EMN-----PSFISDE---------------------------------TPNKDINVSFYN    113
AtMCM6              EMN-----PSFISDD---------------------------------TPNKDINVSFYN    113
BrMCM6              EMN-----PSFVSDE---------------------------------TPNKDINVSFYN    113
ZmMCM6              EHRAGENRAPLISDD---------------------------------SPNKDINIAFYN    118
PsMCM6              ELK-----PTFISDD---------------------------------NPNKDINVAFYN    112
MCM6_Glyma09g05240  DLK-----PSIVSDD---------------------------------SPDKDINIAFYN    113
MCM6_Glyma15g16570  DLK-----PSLVSDD---------------------------------SPDKDINIAFYN    113
AtMCM2              DLH-----PNYKNI------------------------------------HTKIYVRVTN    313
BoMCM2_1            DLH-----PNYKNI------------------------------------HQKIYVRVTN    301
BrMCM2_1            DLH-----PNYKNI------------------------------------HQKIYVRVTN    304
BoMCM2_2            DLH-----PNYKNI------------------------------------HQKIYVRVTN    299
BrMCM2_2            DLH-----PNYKNI------------------------------------HQKIYVRVTN    308
ZmMCM2              DLH-----KNYRNI------------------------------------HQKIYVRISN    334
PsMCM2              QLH-----PNYKHI------------------------------------HQKIYVRITN    314
MCM2_Glyma07g36680  ELH-----PNYRNI------------------------------------HQKIYVRITN    307
MCM2_Glyma17g03920  ELH-----PNYRNI------------------------------------HQKIYVRITN    313
ZmMCM5              SLR-----SKVAGET----------------------------GEMEEPVTGDVQIFLSS    131
AtMCM5              GLK-----MREANEG----------------------------GVMEEPLTRDVQILLTS    127
BoMCM5              GLR-----MREANET----------------------------GEMEEPTPSDVQILLTS    127
BrMCM5              GLR-----MREANET----------------------------GEMEEPTPSDVQILLTS    127
PsMCM5              NLK-----TKVAGDT----------------------------GVMEDAAPGDVQILLTS    130
MCM5_Glyma13g22420  NLK-----TKVAGDT----------------------------GDMEDQTPGDVQILLTS    132
MCM5_Glyma17g11220  NLK-----TKVAGDT----------------------------GDMEDQTPGDVQILLTS    132
ZmMCM3              NLD-----PKFLKEGER-----------------------VMV-----GFSG------PF    102
AtMCM3              AID-----PKYLKEGEL-----------------------VLV-----GFEG------YF    97
BrMCM3_1            NID-----PKYLKEGEQ-----------------------VLV-----GFEG------HF    99
BoMCM3              NID-----PKYLKEGEQ-----------------------VLV-----GFEG------HF    99
BrMCM3_2            NID-----PKYLKEGEQ-----------------------VLV-----GFEG------HF    99
MCM3_Glyma05g25980  AID-----PKYLKEGEQ-----------------------VLV-----GFEG------PF    101
MCM3_Glyma08g08920  AID-----PKYLKEGEQ-----------------------VLV-----GFEG------PF    101
PsMCM3              AID-----PKYLKEGEH-----------------------VLV-----GLEG------PF    34
AtMCM7              EPT-----EAFP-DDDHDILMTQRADDGTD-NPDVSDPHQQIPSEIKRYYEVYFK--APS    136
BoMCM7_2            EPT-----EAFP-DDDHDILMTQRADDGTD-NADVSDPRQQIPSEIKRFYEVYFK--APS    138
BrMCM7_1            EPT-----EAFP-DDDHDILMTQRADDGTD-NADVSDPRQQIPSEIKRFYEVYSK--APS    138
BoMCM7_1            EPT-----EAFP-DDDHDILMTQRADEGAD-NADVSDPRQQIPSEIKRFYEVYFK--APS    138
BrMCM7_2            EPT-----EAFP-DDDHDILMTQRADDGAD-NADVSDPRQQIPSEIKRFYEVYFK--APS    138
ZmMCM7              EPT-----EAYTVDEDQDILMTQRVDEGADGGADGTDPLQKMPPEIKRFFEVYIK--AFS    138
PsMCM7              EPT-----EAFI-DDDHDILMTQRSDEGTE-GADGSDPHQKMPSEIKRFFEVYVK--ASS    138
MCM7_Glyma03g37770  EPT-----EDFT-DDDHDILMTQRSDEGVE-GTDGSDPRQKMPPEIKRYYELYIK--ASS    138
MCM7_Glyma19g40370  EPT-----EDFT-DDDHDILMTQRSDEGAE-GTDGSDPRQKMPAEIKRYYELYIK--ASS    138
ZmMCM4              --------------------------------------LMDLVARIEPLFEKHIQTRIYN    235
AtMCM4              --------------------------------------LMDIVSTINRLFEKHVQVRIFN    229
BoMCM4              --------------------------------------LMDIVSSIDRLFEKHVQVRIFN    213
BrMCM4              --------------------------------------LMDIVSSIDRLFEKHVQVRIFN    213
MCM4_Glyma11g12110  --------------------------------------LMNMVGELKPMFEKHIQTRIFN    221
MCM4_Glyma12g04320  --------------------------------------LMNMVSELKPMFEKHIQTRIFN    225
PsMCM4              --------------------------------------LMNMVTRMKPMFEKHVQTRIFN    218


BoMCM6_2            LPFTKRLRELTTAEIGKLVSVTGVVTRTSEVRPELLYGTFKCLDCGSVIKNVEQQ--FKY    74
BoMCM6_1            LPFTKRLRELTTSEIGKLVSVTGVVTRTSEVRPELLYGTFKCLDCGSVIKNVEQQ--FKY    171
AtMCM6              LPFTKRLRELTTAEIGKLVSVTGVVTRTSEVRPELLYGTFKCLDCGSVIKNVEQQ--FKY    171
BrMCM6              LPFTKRLRELTTSEIGKLVSVTGVVTRTSEVRPELLYGTFKCLDCGSVIKNVEQQ--FKY    171
ZmMCM6              IPMLKKLRELGTAEIGKLTSVMGVVTRTSEVRPELLQGTFKCLDCGNVVKNVEQQ--FKY    176
PsMCM6              IPIVKRLRELATSEIGRLVSVTGVVTRTSEVRPELLQGTFKCLECGGVIKNVEQQ--FKY    170
MCM6_Glyma09g05240  MPIVKRLRELGTSEIGRLVSVTGVVTRTSEVRPELLQGTFKCLECGGVIKNVEQQ--FKY    171
MCM6_Glyma15g16570  MPIVKRLRELGTSEIGRLVSVTGVVTRTSEVRPELLHGTFKCLECGGVIKNVEQQ--FKY    171
AtMCM2              LPVNDQIRNIRQIHLNTMIRIGGVVTRRSGVFPQLQQVKYDCNKCGAVLGPFFQNS-YSE    372
BoMCM2_1            LPVNDQIRNIRQIHLNTMIRIGGVVTRRSGVFPQLQQVKYDCNKCGAILGPFFQNS-YSE    360
BrMCM2_1            LPVNDQIRNIRQIHLNTMIRIGGVVTRRSGVFPQLQQVKYDCNKCGAILGPFFQNS-YSE    363
BoMCM2_2            LPVNDQIRNIRQIHLNTMIRIGGVVTRRSGVFPQLQQVKYDCNKCGAVLGPFFQNS-YSE    358
```

```
BrMCM2_2               LPVNDQIRNIRQIHLNTMIRIGGVVTRRSGVFPQLQQVKYDCNKCGAVLGPFFQNS-YSE   367
ZmMCM2                 LPVYDQIRNIRQIHLNTMIRIGGVVTRRSGVFPQLQQVKYDCNKCGTILGPFFQNS-YTE   393
PsMCM2                 LPVYDQIRNIRQIHLNTMIRIGGVVTRRSGVFPQLQQVKYGCSKCGAILGPFFQNS-YSE   373
MCM2_Glyma07g36680     LPVYDQIRNIRQIHLNTMIRIGGVVTRRSGVFPQLQQVKYDCNKCGAILGPFFQNS-YSE   366
MCM2_Glyma17g03920     LPVYDQIRNIRQIHLNTMIRIGGVVTRRSGVFPQLQQVKYDCNKCGAILGPFFQNS-YSE   372
ZmMCM5                 KENCLSMRSVGADYMSKLVKIAGIAIAASRVKAKATHVTLICKNCRSVRTVPCRPGLGGA   191
AtMCM5                 REDPVSMRLLGAQYISKLVKISGISIAASRVKAKATYVFLVCKNCKKTREVPCRPGLGGA   187
BoMCM5                 REDPVSMRLLGAQYISKLVKISGISIAASRVKAKATYVFLVCKNCRKTREVPCRPGLGGA   187
BrMCM5                 REDPVSMRLLGAQYISKLVKISGISIAASRVKAKATYVFLVCKNCRKTREVPCRPGLGGA   187
PsMCM5                 KEDSLSMRSLGAQYISKLVKIAGITIAASRTKAKATYVTLICKNCKKGKQVPCRPGLGGA   190
MCM5_Glyma13g22420     KEDPVSMRSLGAQYISKLVKIAGITIAASRTKAKATYVTLICKNCKKGKQVPCRPGLGGA   192
MCM5_Glyma17g11220     KEDPVSMRSLGAQYISKLVKIAGITIAASRTKAKATYVTLICKNCKKGKQVPCRPGLGGA   192
ZmMCM3                 GFHRVTPRDLMSSFIGTMVCVEGIVTKCSLVRPKVVKSVHFCPVTGDFLSREYRD-ITSF   161
AtMCM3                 VSRVVTPRELLSDFIGSMVCVEGIVTKCSLVRPKVVKSVHFCPSTGEFTNRDYRD-ITSH   156
BrMCM3_1               VSRRVTPRELLSEFIGSLVCVEGIVTKCSLVRPKVVKSVHFCPSTGEFTNREYRD-ITSH   158
BoMCM3                 VSRRVTPRELLSEFIGSLVCVEGIVTKCSLVRPKVVKSVHFCPSTGEFTNREYRD-ITSH   158
BrMCM3_2               VSRRVTPRELLSEFIGSLVCVEGIVTKCSLVRPKVVKSVHFCPSTGEFTNREYRD-ITSH   158
MCM3_Glyma05g25980     VSRRVTPRDLLSQFIGSMVCVEGIVTKCSLVRPKVVKSVHFCPTTGSFTSREYRD-ITSN   160
MCM3_Glyma08g08920     VSRRVTPRELLSEFIGSMVCIEGIVTKCSLVRPKVVKSVHFCPTTGSFTSREYRD-ITSN   160
PsMCM3                 VSRRVTPRDLLSEFIGSMVCVEGIITKCSLVRPKVVKSVHFCPTTGSFTSRDYRD-ITSN   93
AtMCM7                 KGRPSTIREVKASHIGQLVRISGIVTRCSDVKPLMAVAVYTCEDCGHEIYQEVTS-RV-F   194
BoMCM7_2               KGRPSTIREVKASHIGQLVRIAGIVTRCSDVKPLMAVAVYTCEDCGHEIYQEVTS-RV-F   196
BrMCM7_1               KGRPSTIREVKASHIGQLVRIAGIVTRCSDVKPLMAVAVYTCEDCGHEIYQEVTS-RV-F   196
BoMCM7_1               KGRPSTIREVKASHIGQLVRIAGIVTRCSDVKPLMAVAVYTCEDCGHEIYQEVTS-RV-F   196
BrMCM7_2               KGRPSTIREVKASHIGQLVRIAGIVTRCSDVKPLMAVAVYTCEDC-------VTS-RV-F   189
ZmMCM7                 KVTPLTIRQVKASNIGQLVKISGIVTRCSDVKPLMQVAVYTCEECGFEIYQEVTA-RV-F   196
PsMCM7                 KGRPFTIREVKASNIGQLVRLAGIVTRCSDVKPLMQVAVYTCEDCGFEIYQEVTA-RI-F   196
MCM7_Glyma03g37770     KGRPSTIREVKASNIGQLVRISGIVIRCSDVKPLMKVAVYTCEDCGFEIYQEVTA-RV-F   196
MCM7_Glyma19g40370     KGRPSTIREVKALNIGQLVRISGIVTRCSDVKPLMKVAVYTCEDCGFEIYQEVTA-RV-F   196
ZmMCM4                 LKSSICLRNLNPSDIEKMVSIKGMIIRCSSVIPELKEAVFRCLVCGFYSEPVMVD-RGRV   294
AtMCM4                 LRTSTSMRNLNPSDIEKMISLKGMIIRSSSIIPEIREAVFRCLVCGYFSDPIIVD-RGKI   288
BoMCM4                 LRSSTSMRNLNPSDIEKMISLKGMIIRSSSIIPEIREAVFRCLVCGYFSDPIIVD-RGKI   272
BrMCM4                 LRSSTSMRNLNPSDIEKMISLKGMIIRSSSIIPEIREAVFRCLVCGYFSDPIIVD-RGKI   272
MCM4_Glyma11g12110     LRNSTSMRNLNPSDIERMVSLKGMVIRSSSIIPEIREAIFRCLVCGFCSEPVPVE-RGRI   280
MCM4_Glyma12g04320     LRTSTSMRNLNPSDIERMVSLKGMVIRSSSIIPEIREAIFRCLVCGFCSEPVPVE-RGRI   284
PsMCM4                 LKTSTSMRNLNPSDVERMISMKGMIIRSSSIIPEIREAIFRCLVCGYCSDPVLVE-RGRI   277
                              * :    :  :  : *:    *            *

BoMCM6_2               TQP------TICVSPTCLNR----ARWALLRQESKFADWQRVRMQETSKEIPAGSLPRSL   124
BoMCM6_1               TQP------TICVSPTCLNR----ARWALLRQESKFADWQRVRMQETSKEIPAGSLPRSL   221
AtMCM6                 TQP------TICVSPTCLNR----ARWALLRQESKFADWQRVRMQETSKEIPAGSLPRSL   221
BrMCM6                 TQP------TICVSPTCLNR----ARWALLRQESKFADWQRVRMQETSKEIPAGSLPRSL   221
ZmMCM6                 TEP------IICVNATCQNR----TKWALLRQESKFTDWQRVRMQETSKEIPAGSLPRSL   226
PsMCM6                 TEP------TICPNATCNNR----TRWALLRQESKFTDWQRVRMQETSKEIPAGSLPRSL   220
MCM6_Glyma09g05240     TEP------TICTNATCSNR----TRWVLLRQESKFADWQRVRMQETSKEIPAGSLPRSL   221
MCM6_Glyma15g16570     TEP------TICANATCSNR----TRWVLLRQESKFADWQRVRMQETSKEIPAGSLPRSL   221
AtMCM2                 V--------KVGSCSECQSK----GPFTVNVEQTIYRNYQKLTIQESPGTVPAGRLPRHK   420
BoMCM2_1               V--------KVGSCSECQSK----GPFTVNVEQTIYRNYQKLTIQESPGTVPAGRLPRHK   408
BrMCM2_1               V--------KVGSCSECQSK----GPFTVNVEQTIYRNYQKLTIQESPGTVPAGRLPRHK   411
BoMCM2_2               V--------KVGSCSECQSK----GPFTVNVEQTIYRNYQKLTIQESPGTVPAGRLPRHK   406
BrMCM2_2               V--------KVGSCSECQSK----GPFTVNVEQTIYRNYQKLTIQESPGTVPAGRLPRHK   415
ZmMCM2                 V--------KVGSCPECQSK----GPFTVNVEQTIYRNYQKLTLQESPGIVPAGRLPRYK   441
PsMCM2                 V--------KVGSCPECQSK----GPFTVNIEQTIYRNFQKLTLQESPGIVPAGRLPRYK   421
MCM2_Glyma07g36680     V--------KVGSCPECQSK----GPFTVNIEQTIYRNFQKLTLQESPGIVPAGRLPRYK   414
MCM2_Glyma17g03920     V--------KVGSCPECQSK----GPFTVNIEQTIYRNFQKLTLQESPGIVPAGRLPRYK   420
ZmMCM5                 IVPRSCDHVPQPGEEPCPLD-----PWIAVPDKSKYVDLQTLKLQENPEDVPTGELPRNV   246
AtMCM5                 IVPRSCDNIPQPGEEPCPLD-----PWMVVPDRSQYVDQQTLKLQENPEDVPTGELPRNM   242
BoMCM5                 IVPRSCDHVPQPGEEPCPLD-----PWMVVPDRSQYVDQQTLKLQENPEDVPTGELPRNM   242
BrMCM5                 IVPRSCDHVPQPGEEPCPLD-----PWMVVPDRSQYVDQQTLKLQENPEDVPTGELPRNM   242
PsMCM5                 VVPRSCDHVPQPGEEPCPID-----PWLIVPDKSKYVDQQTLKLQENPEDVPTGELPRNL   245
MCM5_Glyma13g22420     IVPRSCDHVPQPGEEPCPID-----PWLVVPDKSRYVDQQTLKMQENPEDVPTGELPRNL   247
```

```
MCM5_Glyma17g11220    IVPRSCDHVPQPGEEPCPID-----PWLVVPDKSRYVDQQTLKMQENPEDVPTGELPRNL    247
ZmMCM3                VGL------PTGS--VYPTRDDNGNLLVTEYGMCEYKDHQTLSMQEVPENSAPGQLPRTV    213
AtMCM3                AGL------PTGS--VYPTRDDKGNLLVTEYGLCKYKDHQTLSIQEVPENAAPGQLPRSV    208
BrMCM3_1              AGL------PTGS--VYPTRDDNGNLLVTEYGLCKYKDHQTMSIQEVPENAAPGQLPRSV    210
BoMCM3                AGL------PTGS--VYPTRDDNGNLLVTEYGLCKYKDHQTLSIQEVPENAAPGQLPRSV    210
BrMCM3_2              AGL------PTGS--VYPTRDDNGNLLVTEYGLCKYKDHQTLSIQEVPENAAPGQLPRSV    210
MCM3_Glyma05g25980    LGL------PTGS--VYPTRDENGNLLVTEFGLCKYKDHQTLSIQEVPENSAPGQLPRTV    212
MCM3_Glyma08g08920    LGL------PTGS--VYPTRDENGNLLVTEFGLCKYKDHQTLSIQEVPENSAPGQLPRTV    212
PsMCM3                LGL------PTGS--VYPTRDENGNLLVTEYGLCKYKDHQTLSMQEVPENSAPGQLPRTV    145
AtMCM7                MPL------FKCPSSRCRLN-SKAGNPILQLRASKFLKFQEAKMQELAEHVPKGHIPRSM    247
BoMCM7_2              MPL------FKCPSSRCRVN-SKAGNPILQLRASKFLKFQEAKMQELAEHVPKGHIPRSM    249
BrMCM7_1              MPL------FKCPSSRCRVN-SKAGNPILQLRASKFLKFQEAKMQELAEHVPKGHIPRSM    249
BoMCM7_1              MPL------FKCPSSRCRVN-SKAGNPILQLRASKFLKFQEAKMQELAEHVPKGHIPRSM    249
BrMCM7_2              MPL------FKCPSSRCRVN-GKSGNPILQLRASKFLKFQEAKMQELAEHVPKGHIPRSM    242
ZmMCM7                MPL------IECPSQRCKLN-KAKGNLILQLRASKFLKFQEVKLQELAEHVPKGHIPRAL    249
PsMCM7                MPL------FECPSRRCVMN-KNKGNVILQLRASKFLRFQEAKIQELAEHVPKGHIPRTM    249
MCM7_Glyma03g37770    MPL------FECPSKRCDTN-RRKGNVILQLRASKFLRFQEAKIQELAEHVPKGHIPRTM    249
MCM7_Glyma19g40370    MPL------FECPSKRCDTN-RRKGNVILQHRASKFLRFQEAKIQELAEHVPKGHIPRTM    249
ZmMCM4                TEP------HICQKEQCKAT----NSMTLVHNRCRFSDKLIIKLQETPDEIPEGGTPHTV    344
AtMCM4                SEP------PTCLKQECMTK----NSMTLVHNRCRFADKQIVRLQETPDEIPEGGTPHTV    338
BoMCM4                SEP------PTCLKQECLAK----NSMTLVHNRCRFADKQIVRLQETPDEIPEGGTPHTV    322
BrMCM4                SEP------PTCLKQECLAK----NSMTLVHNRCRFADKQIVRLQETPDEIPEGGTPHTV    322
MCM4_Glyma11g12110    TEP------TICLKEECQSR----NSMTLVHNRCRFADKQIVRVQETPDEIPEGGTPHTV    330
MCM4_Glyma12g04320    TEP------TICLREECQSR----NSMALVHNRCRFADKQIVRVQETPDEIPEGGTPHTV    334
PsMCM4                AEP------TVCLREECQSR----NSMTLVHNRCKFTDKQIVRLQETPDEIPEGGTPHTV    327
                                                         :       :**       *  *:

BoMCM6_2              DVILRHEIVEQARAGDTVIFTGAVIVLPDISAMASPGERAECRRDSSQQKSSTAGHEGFK    184
BoMCM6_1              DVILRHEIVEQARAGDTVIFTGTVVVIPDISALAAPGERAECRRDSSQQKSSTAGHEGVK    281
AtMCM6                DVILRHEIVEQARAGDTVIFTGTVVVIPDISALAAPGERAECRRDSSQQKSSTAGHEGVQ    281
BrMCM6                DVILRHEIVEQARAGDTVIFTGTVVVIPDISALAAPGERAECRRDSSQQKSSTAGHEGVK    281
ZmMCM6                DVILRHEIVEKARAGDTVIFTGTVVAVPDVMALTSPGERAECRREAPQRKNG-GVQEGVK    285
PsMCM6                DVILRHEIVEHARAGDTVIFTGTVIVIPDILALASPGERSECRREASQRKGSSSGNEGVR    280
MCM6_Glyma09g05240    DVILRHEIVEQARAGDTVIFTGTVVAIPDIMALASPGERSECRRDASQRRGSTAGNEGVS    281
MCM6_Glyma15g16570    DIILRHEIVEHARAGDTVIFTGTVVVIPDIMALASPGERSECRRDASQRKGSTAGNEGVS    281
AtMCM2                EVILLNDLIDCARPGEEIEVTGIYTNNFDLSLN--T--------------------KNGF    458
BoMCM2_1              EVILLNDLIDCARPGEEIEVTGIYTNNFDLSLN--T--------------------KNGF    446
BrMCM2_1              EVILLNDLIDCARPGEEIEVTGIYTNNFDLSLN--T--------------------KNGF    449
BoMCM2_2              EVILLNDLIDCARPGEEIEVTGIYTNNFDLSLN--T--------------------KNGF    444
BrMCM2_2              EVILLNDLIDCARPGEEIEVTGIYTNNFDLSLN--T--------------------KNGF    453
ZmMCM2                EVILLNDLIDCARPGEEIEVTGIYTNNFDLSLN--T--------------------KNGF    479
PsMCM2                EVILLNDLIDCARPGEEIEVTGIYTNNFDLSLN--T--------------------KNGF    459
MCM2_Glyma07g36680    EVILLNDLIDCARPGEEIEVTGVYTNNFDLSLN--T--------------------KNGF    452
MCM2_Glyma17g03920    EVILLNDLIDCARPGEEIEVTGVYTNNFDLSLN--T--------------------KNGF    458
ZmMCM5                LLSVDRHLVQTIVPGTRLTVVGIYSVYQAST---TQ--------------------KGAV    283
AtMCM5                LLSVDRHLVQTIVPGTRLTVMGIYSIFQASSSSNSH--------------------KGAV    282
BoMCM5                LLSVDRHLVQLIVPGTRLTVMGIYSIFQASSSSNSH--------------------KGAV    282
BrMCM5                LLSVDRHLVQLIVPGTRLTVMGIYSIFQASSSSNSH--------------------KGAV    282
PsMCM5                LLSVDRHLVQTVVPGPRLTIVGIYSIFQASNSSTSN--------------------KGAV    285
MCM5_Glyma13g22420    LLSVDRHLVQTVVPGSRLTIMGIFSIYQASNSNTSH--------------------KGAV    287
MCM5_Glyma17g11220    LLSLDRHLVQTVVPGSRLTIMGIFSIYQASNSNTSN--------------------KGAV    287
ZmMCM3                DVIVEDDLVDCCKPGDRVSIVGVYKALPGK----SK--------------------GSVS    249
AtMCM3                DVIAEDDLVDSCKPGDRVSVFGIYKALPGK----SK--------------------GSVN    244
BrMCM3_1              DVIAEDDLVDSCKPGDRVAVVGIYKALPGK----SK--------------------GSVN    246
BoMCM3                DVIAEDDLVDSCKPGDRVAIVGIYKALPGK----SK--------------------GSVN    246
BrMCM3_2              DVIAEDDLVDSCKPGDRVAIVGIYKALPGK----SK--------------------GSVN    246
MCM3_Glyma05g25980    DVIAEDDLVDSCKPGDRVAIVGIYKALAGK----SK--------------------GSVN    248
MCM3_Glyma08g08920    DVIAEDDLVDSCKPGDRVAIVGIYKALAGK----SK--------------------GSVN    248
PsMCM3                DVIAEDDLVDSCKPGDRVAIVGIYKALPGK----SK--------------------GSVN    181
AtMCM7                TVHLRGELTRKVSPGDVVEFSGIFLPIPYTGFK--A--------------------LRAG    285
BoMCM7_2              TVHLRGELTRKVAPGDVVEFSGIFLPIPYTGFK--A--------------------LRAG    287
```

```
BrMCM7_1              TVHLRGELTRKVAPGDVVEFSGIFLPIPYTGFK--A--------------------LRAG   287
BoMCM7_1              TVHLRGELTRKVAPGDVVEFSGIFLPIPYTGFK--A--------------------LRAG   287
BrMCM7_2              TVHLRGELTRKVAPGDVVEFSGIFLPIPYTGFK--A--------------------LRAG   280
ZmMCM7                TVHLRGELTRKVAPGDVVEMSGIFLPMPYYGFR--A--------------------MRAG   287
PsMCM7                TVHLRGELTRKVAPGDVVELSGIFLPIPYVGFR--A--------------------MRAG   287
MCM7_Glyma03g37770    TVHLRGELTRKVAPGDVVEFSGIFLPIPYTGFR--A--------------------MRAG   287
MCM7_Glyma19g40370    TVHLRGELTRKVAPGDVVELSGIFLPIPYTGFR--A--------------------MRAG   287
ZmMCM4                SVLMHDKLVDAGKPGDRVEITGIYRAMSIRIGP-TQ--------------------RTVK   383
AtMCM4                SLLLHDKLVDNGKPGDRIEVTGIYRAMTVRVGP-AH--------------------RTVK   377
BoMCM4                SLLLHDKLVDNGKPGDRIEVTGIYRAMTVRVGP-AH--------------------RT--   359
BrMCM4                SLLLHDKLVDNGKPGDRIEVTGIYRAMTVRVGP-AH--------------------RT--   359
MCM4_Glyma11g12110    SLLMHDKLVDTAKPGDRVEVTGIYRAMSVRIGP-TQ--------------------RTVK   369
MCM4_Glyma12g04320    SLLMHDKLVDNAKPGDRVEVTGIYRAMSVRVGP-TQ--------------------RTVK   373
PsMCM4                SLLMHDKLVDTGKPGDRVEVTGIYRAMSVRVGP-TQ--------------------RSVK   366
                       :    .:      *  : . *

BoMCM6_2              GLKALGVRDLSYRLSFIANSVQIADGSRNTD---TRHRQNDSNEDDQQQFTGEELYEIQL   241
BoMCM6_1              GLKALGVRDLSYRLAFIANSVQIADGSRNTD---MRNRQNDSNEDDQQQFTGEELDEIQQ   338
AtMCM6                GLKALGVRDLSYRLAFIANSVQIADGSRNTD---MRNRQNDSNEDDQQQFTAEELDEIQQ   338
BrMCM6                GLKALGVRDLSYRLAFIANSVQIADGSRNTD---MRNRQNDSNEDDQQQFTGEELDEIQQ   338
ZmMCM6                GLKSLGVRDLSYRLAFVANSVQVADGRREVD---IRERDTDGDDSERQKFTEEEEDEVVR   342
PsMCM6                GLRALGVRDLSYRLAFIANSVQICDGRREID---IRNRKKDSEED-DLLFSQQELDEVQR   336
MCM6_Glyma09g05240    GLKALGVRDLNYRLAFIANSVQICDGRREID---IRNRKKDADDD-NQQFTDQELEEIKR   337
MCM6_Glyma15g16570    GLKALGVRDLNYRLAFIANSAQICDGRREID---IRNRKKDVDED-NQQFTDQELEEIKR   337
AtMCM2                P---------VFATVVEANYVTK-----------------KQDLFSAYKLTQEDKTQIEE   492
BoMCM2_1              P---------VFATVVEANYVTK-----------------KQDLFSAYKLTQEDKTQIEE   480
BrMCM2_1              P---------VFATVVEANYVTK-----------------KQDLFSAYKLTQEDKTQIEE   483
BoMCM2_2              P---------VFATVVEANYVTK-----------------KQDLFSAYKLTQEDKTQIEE   478
BrMCM2_2              P---------VFATVVEANYVTK-----------------KQDLFSAYKLTQEDKTQIEE   487
ZmMCM2                P---------VFATVVEANYVAK-----------------KQDLFSAYKLTDEDKAEIEK   513
PsMCM2                P---------VFSTVVEANYVTK-----------------KQDLFSAYKLTQEDKEEIEN   493
MCM2_Glyma07g36680    P---------VFATVVEANYVTK-----------------KQDLFSAYKLTQEDIEEIEN   486
MCM2_Glyma17g03920    P---------VFATVVEANYVTK-----------------KQDLFSAYKLTQEDIEEIEN   492
ZmMCM5                G---------VKQPYIRVVGLEQS---------------RDNNSNGPSNFTLDEEMEFKE   319
AtMCM5                A---------IRQPYIRVVGLEDT---------------NEASSRGPANFTPDEEEEFKK   318
BoMCM5                A---------IRQPYIRVVGLEDT---------------NEASSRGPANFTPDEEEEFKK   318
BrMCM5                A---------IRQPYIRVVGLEDT---------------NEASSRGPANFTPDEEEEFKK   318
PsMCM5                A---------VRQPYIRVVGIEDA---------------NEAKSRGPTSFTTEEIEEFKK   321
MCM5_Glyma13g22420    A---------IRQPYIRVVGIEET---------------NETNSRGPAAFTQDEIEEFKK   323
MCM5_Glyma17g11220    A---------IRQPYIRVVGIEET---------------NETNSRGPAAFTQDEIEEFKK   323
ZmMCM3                G---------VFRTVLIANNVSL-----------------LNKEANAPVYTREDLKRMKE   283
AtMCM3                G---------VFRTILIANNIAL-----------------LNKEANAPIYTKQDLDNIKN   278
BrMCM3_1              G---------VFRTILIANNISL-----------------LNKEANAPIYTPRDLQEIKK   280
BoMCM3                G---------VFRTILIANNISL-----------------LNKEANAPIYTPRDLQEIKK   280
BrMCM3_2              G---------VFRTILIANNISL-----------------LNKEANAPIYTPRDLQEIKK   280
MCM3_Glyma05g25980    G---------VFRTVLIANNVSL-----------------LNKEANAPIYSAEDVKSIKE   282
MCM3_Glyma08g08920    G---------VFRTVLIANNVSL-----------------LNKEANAPIYSAEDVKNIKE   282
PsMCM3                G---------VFRTVLIANNVAL-----------------LNKEANAPIYSTEDLKNIKK   215
AtMCM7                L---------VADTYLEATSVTH-----------------FKKKYEEYEFQKDEEEQIAR   319
BoMCM7_2              L---------VADTYLEATSVTH-----------------FKKKYEEYEFQKDEEEQIAR   321
BrMCM7_1              L---------VADTYLEATSVTH-----------------FKKKYEEYEFQKDEEEQIAR   321
BoMCM7_1              L---------VADTYLEATAVTH-----------------FKKKYEEYEFQKDEEEQIAR   321
BrMCM7_2              L---------VADTYLEATAVTH-----------------FKKKYEEYEFQKDEEEQIAR   314
ZmMCM7                L---------VADTYLEAMSVTH-----------------FKKKYEEYDLKGDEQEQIDR   321
PsMCM7                L---------VADTYLEAMSVSH-----------------FKKKYEEYELRGDEEEQIKR   321
MCM7_Glyma03g37770    L---------VADTYLEAMSVMH-----------------FKKKYEEYEFRGDEEEQIAR   321
MCM7_Glyma19g40370    L---------VADTYLEAMSVTH-----------------FKKKYEEYEFRGDEEEQIAR   321
ZmMCM4                S---------IFKTYIDCLHIKKTDKSRLHVEDTMDIDNSNASKSTEEDFLSDKVEKLKE   434
AtMCM4                S---------VFKTYIDCLHIKKASKLRMSAEDPMDVDNSLRRVDEDVELDEEKLRKFQE   428
BoMCM4                -------------TYIDCLHIKKASKTRMAAEDPMDVDNSLRRVDEDVELDEEKLKKFEE   406
BrMCM4                -------------TYIDCLHIKKASKTRMAAEDPMDVDNSLRRVDEDVELDEEKLKKFEE   406
```

```
MCM4_Glyma11g12110      S---------LFKTYIDCLHIKKTDKSRMLVEDAMDVDGQD--KNAEVLFDEEKVAQLKE   418
MCM4_Glyma12g04320      S---------LFKTYIDCLHIKKTDKSRMFVEDVMDVDGQD--RNAEVLFDEEKVAQLKE   422
PsMCM4                  S---------LFKTYIDCLHIKKTSKSRMLVEDAMEADSGQGRNAEEVIFSEEKVAQLRE   417
                                       .                                     .   .

BoMCM6_2                MKKTPDYFNKLVGSMAPTVFRHQDIKRAILLMLLGGVHKTTTHEGINLRGDINVCIVGDP   301
BoMCM6_1                MRNTPDYFNKLVGSMAPTVFGHQDIKRAVLLMLLGGVHKT-THEGINLRGDINVCIVGDP   397
AtMCM6                  MRNTPDYFNKLVGSMAPTVFGHQDIKRAVLLMLLGGVHKT-THEGINLRGDINVCIVGDP   397
BrMCM6                  MRNTPDYFNKLVGSMAPTVFGHQDIKRAVLLMLLGGVHKT-THEGINLRGDINVCIVGDP   397
ZmMCM6                  MRNTPDFFNKIVDSICPTVFGHQEIKRAVLLMLLGGVHKI-THEGINLRGDINVCIVGDP   401
PsMCM6                  MRNTPDFFTKLVESVAPTIFGHQDIKRAILLMLMSGVHKS-THEGISLRGDINVCIVGDP   395
MCM6_Glyma09g05240      MRSTPDFFTKLVESIAPTVFGHPDIKRAILLMLLGGVHKF-THEGINLRGDINVCVVGDP   396
MCM6_Glyma15g16570      MRSTPDFFTKLVESIAPTVFGHPDIKRAILLMLLGGVHKF-THEGINLRGDINVCVVGDP   396
AtMCM2                  LSKDPRIVERIIKSIAPSIYGHEDIKTALALAMFGGQEKN-IKGKHRLRGDINVLLLGDP   551
BoMCM2_1                LSKDPRIVERIIKSIAPSIYGHEDIKTAIALAMFGGQEKN-IKGKHRLRGDINVLLLGDP   539
BrMCM2_1                LSKDPRIVERIIKSIAPSIYGHEDIKTAIALAMFGGQEKN-IKGKHRLRGDINVLLLGDP   542
BoMCM2_2                LSKDPRIVERIIKSIAPSIYGHEDIKTAIALAMFGGQEKN-IKGKHRLRGDINVLLLGDP   537
BrMCM2_2                LSKDPRIVERIIKSIAPSIYGHEDIKTAIALAMFGGQEKN-IKGKHRLRGDINVLLLGDP   546
ZmMCM2                  LSKDPRIGERIIKSIAPSIYGHEDIKTAIALAMFGGQEKN-VKGKHRLRGDINVLLLGDP   572
PsMCM2                  LGKDPRIGERIVKSIAPSIYGHDDIKTGIALAMFGGQEKN-VEGKHRLRGDINVLLLGDP   552
MCM2_Glyma07g36680      LAKDPRIGERIVKSIAPSIYGHDDIKTAIALAIFGGQEKN-VEGKHRLRGDINVLLLGDP   545
MCM2_Glyma17g03920      LAKDPRIGERIVKSIAPSIYGHDDIKTAIALAMFGGQEKN-VEGKHRLRGDINVLLLGDP   551
ZmMCM5                  FAQRPDAYAKLCSMIGPSIYGHSDVKKAIACLLFGGSKKR-LPDGVRLRGDIHVLLLGDP   378
AtMCM5                  FADSQDVYKNICTKIAPSIFGHEDVKRAAACLLFGGSRKS-LPDGVKLRGDINVLLLGDP   377
BoMCM5                  FAGGQDVYSNICTKIAPSIFGHEDVKKAVACLLFGGSRKS-LPDGVKLRGDINVLLLGDP   377
BrMCM5                  FAASQDVYSNICTKIAPSIFGHEDVKKAVACLLFGGSRKS-LPDGVKLRGDINVLLLGDP   377
PsMCM5                  FASEPDAYKNICSKIAPSIFGHEDVKKAVACLLFGGSRKH-LPDGVRLRGDINVLLLGDP   380
MCM5_Glyma13g22420      FASEPDAYKNICSMIAPSIFGHEEVKKAVACLLFGGSRKN-LPDGVRLRGDINVLLLGDP   382
MCM5_Glyma17g11220      FAAEPDAYKNICSMIAPSIFGHDDVKKAVACLLFGGSRKN-LPDGVRLRGDINVLLLGDP   382
ZmMCM3                  ISRRNDTFDLLGNSLAPSIYGHLWIKKAVVLLMLGGVEKN-LKNGTHLRGDINMMMVGDP   342
AtMCM3                  IARRDDAFDLLARSLAPSIYGHAWIKKAVVLLMLGGVEKN-LKNGTHLRGDINMMMVGDP   337
BrMCM3_1                IAARDDAFDLLSRSLAPSIYGHAWIKKAVILLMLGGMEKN-LKNGTHLRGDINMMMVGDP   339
BoMCM3                  IAARDDAFDLLSRSLAPSIYGHAWIKKAVILLMLGGMEKN-LKNGTHLRGDINMMMVGDP   339
BrMCM3_2                IAARDDAFDLLSRSLAPSIYGHAWIKKAVILLMLGGMEKN-LKNGTHLRGDINMMMVGDP   339
MCM3_Glyma05g25980      IAARDDAFDLLSNSLAPSIYGHSWIKKAVILLMLSGVEKN-LKNGTHLRGDINMMMVGDP   341
MCM3_Glyma08g08920      IAARDDAFDLLSNSLAPSIYGHSWIKKAVVLLMLSGVEKN-LKNGTHLRGDINMMMVGDP   341
PsMCM3                  IAERDDTFDLLGNSLAPSIHGHSWIKKAVILLMLSGVEKN-LKNGTHLRGDINMMMVGDP   274
AtMCM7                  LAEDGDIYNKLSRSLAPEIYGHEDIKKALLLLLVGAPHRQ-LKDGMKIRGDVHICLMGDP   378
BoMCM7_2                LAEDGDIYNKLSRSLAPEIYGHEDIKKALLLLLVGAPHRQ-LKDGMKIRGDVHICLMGDP   380
BrMCM7_1                LAEDGDIYNKLSRSLAPEIYGHEDIKKALLLLLVGAPHRQ-LKDGMKIRGDVHICLMGDP   380
BoMCM7_1                LAEDGDIYNKLSRSLAPEIYGHEDIKKALLLLLVGAPHRQ-LKDGMKIRGDVHICLMGDP   380
BrMCM7_2                LAEDGDIYNKLSRSLAPEIYGHEDIKKALLLLLVGAPHRQ-LKDGMKIRGDVHICLMGDP   373
ZmMCM7                  LAEDGDIYSKLARSLAPEIFGHEDVKKALLLLLVGAPHRK-LADGMKIRGDLHICMMGDP   380
PsMCM7                  LAEDGDIYDKLARSLAPEIFGHEDIKKALLLLLVGAPHRQ-LKDGMKIRGDLHICLMGDP   380
MCM7_Glyma03g37770      LAEDGDIYNKLARSLAPEIFGHDDIKKALLLLLVGAPHRT-LKDGMKIRGDLHICLMGDP   380
MCM7_Glyma19g40370      LAEDGDIYNKLSRSLAPEIFGHDDIKKALLLLLVGAPHRT-LNDGMKIRGDLHICLMGDP   380
ZmMCM4                  LSKLPDIYERLTRSLAPNIWELDDVKRGLLCQLFGGNPLK-LPSGASFRGDINILLVGDP   493
AtMCM4                  LSKQPDIYERLSRSLAPNIWELDDVKKGLLCQLFGGNALN-LASGANFRGDINILLVGDP   487
BoMCM4                  LSKQPDIYEKLARSLAPNIWELDDVKKGLLCQLFGGNALN-LASGANFRGDINILLVGDP   465
BrMCM4                  LSKQPDIYEKLARSLAPNIWELDDVKKGLLCQLFGGNALN-LASGANFRGDINILLVGDP   465
MCM4_Glyma11g12110      LSKRPDIYEILTKSMAPNIWELDDVKKGLLCQLFGGNALK-LASGANFRGDINILLVGDP   477
MCM4_Glyma12g04320      LSKRPDIYEILTNSLAPNIWELDDVKKGLLCQLFGGNALK-LASGANFRGDINILLVGDP   481
PsMCM4                  LSKQPDIYERLTKSLAPNIWELDDVKKGLLCQLFGGNALK-LATGASFRGDINVLLVGDP   476
                        :         :   : * :     :* .    :...           :***::: ::***

BoMCM6_2                SCAKSQFLKYTAGIVPRSVYTSGKSSSAAGLTATVAKEPETGEFCIEAGALMLADNGICC   361
BoMCM6_1                SCAKSQFLKYTASIVPRSVYTSGKSSSAAGLTATVAKEPETGEFCIEAGALMLADNGICC   457
AtMCM6                  SCAKSQFLKYTAGIVPRSVYTSGKSSSAAGLTATVAKEPETGEFCIEAGALMLADNGICC   457
BrMCM6                  SCAKSQFLKYTAGIVPRSVYTSGKSSSAAGLTATVAKEPETGEFCIEAGALMLADNGICC   457
ZmMCM6                  SCAKSQFLKYTAGIVPRSVYTSGKSSSAAGLTATVAKEPETGEFCIEAGALMLADNGVCC   461
PsMCM6                  SCAKSQFLKYTSSIVPRSVYTSGKSSSAAGLTATVAKEPETGEFCIEAGALMLADNGICC   455
```

```
MCM6_Glyma09g05240    SCAKSQFLKYTSGIVPRSVYTSGKSSSAAGLTATVAKEPETGEFCIEAGALMLADNGICC   456
MCM6_Glyma15g16570    SCAKSQFLKYTSGIVPRSVYTSGKSSSAAGLTATVAKEPETGEFCIEAGALMLADNGICC   456
AtMCM2                GTAKSQFLKYVEKTGQRAVYTTGKGASAVGLTAAVHKDPVTREWTLEGGALVLADRGICL   611
BoMCM2_1              GTAKSQFLKYVEKTGQRAVYTTGKGASAVGLTAAVHKDPVTREWTLEGGALVLADRGICL   599
BrMCM2_1              GTAKSQFLKYVEKTGQRAVYTTGKGASAVGLTAAVHKDPVTREWTLEGGALVLADRGICL   602
BoMCM2_2              GTAKSQFLKYVEKTGQRAVYTTGKGASAVGLTAAVHKDPVTREWTLEGGALVLADRGICL   597
BrMCM2_2              GTAKSQFLKYVEKTGQRAVYTTGKGASAVGLTAAVHKDPVTREWTLEGGALVLADRGICL   606
ZmMCM2                GTAKSQFLKYVEKTGHRAVYTTGKGASAVGLTAAVHKDPVTREWTLEGGALVLADRGICL   632
PsMCM2                GTAKSQFLKYVEKTGQRAVYTTGKGASAVGLTAAVHKDPVTREWTLEGGALVLADRGICL   612
MCM2_Glyma07g36680    GTAKSQFLKYVEKTGQRAVYTTGKGASAVGLTAAVHKDPVTREWTLEGGALVLADRGICL   605
MCM2_Glyma17g03920    GTAKSQFLKYVEKTGQRAVYTTGKGASAVGLTAAVHKDPVTREWTLEGGALVLADKGICL   611
ZmMCM5                STAKSQFLKFVEKTAPIAVYTSGKGSSAAGLTASVTRDSSSREFYLEGGAMVLADGGVVC   438
AtMCM5                STAKSQFLKFVEKTAPIAVYTSGKGSSAAGLTASVIRDSSTREFYLEGGAMVLADGGVVC   437
BoMCM5                STAKSQFLKFVEKTAPIAVYTSGKGSSAAGLTASVIRDSSTREFYLEGGAMVLADGGVVC   437
BrMCM5                STAKSQFLKFVEKTAPIAVYTSGKGSSAAGLTASVIRDSSTREFYLEGGAMVLADGGVVC   437
PsMCM5                STAKSQFLKFVEKTAPIAVYTSGKGSSAAGLTASVIQDSSTREFYLEGGAMVLADGGVVC   440
MCM5_Glyma13g22420    STAKSQFLKFVEKTAPIAVYTSGKGSSAAGLTASVIQDSGTREFYLEGGAMVLADGGVVC   442
MCM5_Glyma17g11220    STAKSQFLKFVEKTAPIAVYTSGKGSSAAGLTASVIQDSGTREFYLEGGAMVLADGGVVC   442
ZmMCM3                SVAKSQLLRAVMNIAPLAISTTGRGSSGVGLTAAVTSDQETGERRLEAGAMVLADRGVVC   402
AtMCM3                SVAKSQLLRAIMNIAPLAISTTGRGSSGVGLTAAVTSDQETGERRLEAGAMVLADKGIVC   397
BrMCM3_1              SVAKSQLLRAIMNIAPLAISTTGRGSSGVGLTAAVTSDQETGERRLEAGAMVLADKGIVC   399
BoMCM3                SVAKSQLLRAIMNIAPLAISTTGRGSSGVGLTAAVTSDQETGERRLEAGAMVLADKGIVC   399
BrMCM3_2              SVAKSQLLRAIMNIAPLAISTTGRGSSGVGLTAAVTSDQETGERRLEAGAMVLADKGIVC   399
MCM3_Glyma05g25980    SVAKSQLLRAIMNIAPLAISTTGRGSSGVGLTAAVTSDQETGERRLEAGAMVLADRGVVC   401
MCM3_Glyma08g08920    SVAKSQLLRAIMNIAPLAISTTGRGSSGVGLTAAVTSDQETGERRLEAGAMVLADRGVVC   401
PsMCM3                SVAKSQLLRAIMNIAPLAISTTGRGSSGVGLTAAVTSDQETGERRLEAGAMVLADRGVVC   334
AtMCM7                GVAKSQLLKHIINVAPRGVYTTGKGSSGVGLTAAVMRDQVTNEMVLEGGALVLADMGICA   438
BoMCM7_2              GVAKSQLLKHIINVAPRGVYTTGKGSSGVGLTAAVMRDQVTNEMVLEGGALVLADMGICA   440
BrMCM7_1              GVAKSQLLKHIINVAPRGVYTTGKGSSGVGLTAAVMRDQVTNEMVLEGGALVLADMGICA   440
BoMCM7_1              GVAKSQLLKHIINVAPRGVYTTGKGSSGVGLTAAVMRDQVTNEMVLEGGALVLADMGICA   440
BrMCM7_2              GVAKSQLLKHIINVAPRGVYTTGKGSSGVGLTAAVMRDQVTNEMVLEGGALVLADMGICA   433
ZmMCM7                GVAKSQLLKHIINVAPRGVYTTGRGSSGVGLTAAVQKDPVTNEFVLEGGALVLADMGICA   440
PsMCM7                GVAKSQLLKHIINVAPRGVYTTGKGSSGVGLTAAVQKDPVTNEMVLEGGALVLSDMGICA   440
MCM7_Glyma03g37770    GVAKSQLLKHIINVAPRGVYTTGRGSSGVGLTAAVQKDPVTNEMVLEGGALVLADMGICA   440
MCM7_Glyma19g40370    GVAKSQLLKHIINVAPRGVYTTGRGSSGVGLTAAVQKDPVTNEMVLEGGALVLADMGICA   440
ZmMCM4                GTSKSQLLQYMHKLSPRGIYTSGRGSSAVGLTAYVTKDPETGETVLESGALVLSDKGVCC   553
AtMCM4                GTSKSQLLQYIHKLSPRGIYTSGRGSSAVGLTAYVAKDPETGETVLESGALVLSDRGICC   547
BoMCM4                GTSKSQLLQYIHKLSPRGIYTSGRGSSAVGLTAYVAKDPETGETVLESGALVLSDRGICC   525
BrMCM4                GTSKSQLLQYIHKLSPRGIYTSGRGSSAVGLTAYVAKDPETGETVLESGALVLSDRGICC   525
MCM4_Glyma11g12110    GTSKSQLLQYIHKLSPRGIYTSGRGSSAVGLTAYVTKDPETGETVLESGALVLSDRGICC   537
MCM4_Glyma12g04320    GTSKSQLLQYIHKLSPRGIYTSGRGSSAVGLTAYVTKDPETGETVLESGALVLSDRGICC   541
PsMCM4                GTSKSQLLQYIHKLSPRGIYTSGRGSSAVGLTAYVAKDPETGETVLESGALVLSDRGICC   536
                      . :***:*:        .: *:*:.:*..**** *  :  : *  :*.**::*:* *:

BoMCM6_2              IDEFDKMDIKDQVAINEAMEQQTISITKAGIQATLNARTSILAAANPVGGRYDISKPLKY   421
BoMCM6_1              IDEFDKMDIKDQVAIHEAMEQQTISITKAGIQATLNARTSILAAANPVGGRYDKSKPLKY   517
AtMCM6                IDEFDKMDIKDQVAIHEAMEQQTISITKAGIQATLNARTSILAAANPVGGRYDKSKPLKY   517
BrMCM6                IDEFDKMDIKDQVAIHEAMEQQTISITKAGIQATLNARTSILAAANPVGGRYDKSKPLKY   517
ZmMCM6                IDEFDKMDIKDQVAIHEAMEQQTISITKAGIQATLNARTSILAAANPTGGRYDKSKPLKY   521
PsMCM6                IDEFDKMDIRDQVAIHEAMEQQTISITKAGIQATLNARTSILAAANPAGGRYDKSKPLKY   515
MCM6_Glyma09g05240    IDEFDKMDIRDQVAIHEAMEQQTISITKAGIQATLNARTSILAAANPAGGRYDKSKPLKY   516
MCM6_Glyma15g16570    IDEFDKMDIRDQVAIHEAMEQQTISITKAGIQATLNARTSILAAANPAGGRYDKSKPLKY   516
AtMCM2                IDEFDKMNDQDRVSIHEAMEQQSISISKAGIVTSLQARCSVIAAANPVGGRYDSSKSFAQ   671
BoMCM2_1              IDEFDKMNDQDRVSIHEAMEQQSISISKAGIVTSLQARCSVIAAANPVGGRYDSSKSFAQ   659
BrMCM2_1              IDEFDKMNDQDRVSIHEAMEQQSISISKAGIVTSLQARCSVIAAANPVGGRYDSSKSFAQ   662
BoMCM2_2              IDEFDKMNDQDRVSIHEAMEQQSISISKAGIVTSLQARCSVIAAANPVGGRYDSSKSFAQ   657
BrMCM2_2              IDEFDKMNDQDRVSIHEAMEQQSISISKAGIVTSLQARCSVIAAANPVGGRYDSSKSFAQ   666
ZmMCM2                IDEFDKMNDQDRVSIHEAMEQQSISISKAGIVTSLQARCSVIAAANPVGGRYDSSKTFTQ   692
PsMCM2                IDEFDKMNDQDRVSIHEAMEQQSISISKAGIVTSLQARCSVIAAANPIGGRYDSSKLFTQ   672
MCM2_Glyma07g36680    IDEFDKMNDQDRVSIHEAMEQQSISISKAGIVTSLQARCSVIAAANPVGGRYDSSKTFSQ   665
MCM2_Glyma17g03920    IDEFDKMNDQDRVSIHEAMEQQSISISKAGIVTSLQARCSVIAAANPVGGRYDSSKTFSQ   671
```

```
ZmMCM5               IDEFDKMRPEDRVAIHEAMEQQTISIAKAGITTVLNSRTSVLAAANPIAGRYDDLKTAQD   498
AtMCM5               IDEFDKMRPEDRVAIHEAMEQQTISIAKAGITTVLNSRTSVLAAANPPSGRYDDLKTAQD   497
BoMCM5               IDEFDKMRPEDRVAIHEAMEQQTISIAKAGITTVLNSRTSVLAAANPPSGRYDDLKTAQD   497
BrMCM5               IDEFDKMRPEDRVAIHEAMEQQTISIAKAGITTVLNSRTSVLAAANPPSGRYDDLKTAQD   497
PsMCM5               IDEFDKMRPEDRVAIHEAMEQQTISIAKAGITTVLNSRTSVLAAANPPSGRYDDLKTAQD   500
MCM5_Glyma13g22420   IDEFDKMRPEDRVAIHEAMEQQTISIAKAGITTVLNSRTSVLAAANPPSGRYDDLKTAQD   502
MCM5_Glyma17g11220   IDEFDKMRPEDRVAIHEAMEQQTISIAKAGITTVLNSRTSVLAAANPPSGRYDDLKTAQD   502
ZmMCM3               IDEFDKMNDQDRVAIHEVMEQQTVTIAKAGIHASLNARCSVIAAANPIYGTYDRSLTPTK   462
AtMCM3               IDEFDKMNDQDRVAIHEVMEQQTVTIAKAGIHASLNARCSVVAAANPIYGTYDRSLTPTK   457
BrMCM3_1             IDEFDKMNDQDRVAIYEVMEHQTVTIAKAGIHASLNARCSVVAAANPIYGTYDRSLTPTK   459
BoMCM3               IDEFDKMNDQDRVAIHEVMEQQTVTIAKAGIHASLNARCSVVAAANPIYGTYDRSLTPTK   459
BrMCM3_2             IDEFDKMNDQDRVAIHEVMEQQTVTIAKAGIHASLNARCSVVAAANPIYGTYDRSLTPTK   459
MCM3_Glyma05g25980   IDEFDKMNDQDRVAIHEVMEQQTVTIAKAGIHASLNARCSVVAAANPIYGTYDRSLTPTK   461
MCM3_Glyma08g08920   IDEFDKMNDQDRVAIHEVMEQQTVTIAKAGIHASLNARCSVVAAANPIYGTYDRSLTPTK   461
PsMCM3               IDEFDKMNDQDRVAIHEVMEQQTVTIAKAGIHASLNARCSVVAAANPIYGTYDRSLTPTK   394
AtMCM7               IDEFDKMDESDRTAIHEVMEQQTVSIAKAGITTSLNARTAVLAAANPAWGRYDLRRTPAE   498
BoMCM7_2             IDEFDKMDESDRTAIHEVMEQQTVSIAKAGITTSLNARTAVLAAANPAWGRYDLRRTPAE   500
BrMCM7_1             IDEFDKMDESDRTAIHEVMEQQTVSIAKAGITTSLNARTAVLAAANPAWGRYDLRRTPAE   500
BoMCM7_1             IDEFDKMDESDRTAIHEVMEQQTVSIAKAGITTSLNARTAVLAAANPAWGRYDLRRTPAE   500
BrMCM7_2             IDEFDKMDESDRTAIHEVMEQQTVSIAKAGITTSLNARTSVLAAANPAWGRYDLRRTPAE   493
ZmMCM7               IDEFDKMEESDRTAIHEVMEQQTVSIAKAGITTSLNARTAILAAANPAWGRYDMRRTPAE   500
PsMCM7               IDEFDKMDESDRTSIHEVMEQQTVSIAKAGITTSLNARTAVLAAANPAWGRYDLRRTPAE   500
MCM7_Glyma03g37770   IDEFDKMDESDRTAIHEVMEQQTVSIAKAGITTSLNARTAVLAAANPAWGRYDLRRTPAE   500
MCM7_Glyma19g40370   IDEFDKMDESDRTAIHEVMEQQTVSIAKAGITTSLNARTAVLAAANPAWGRYDLRRTPAE   500
ZmMCM4               IDEFDKMSDNARSMLHEVMEQQTVSIAKAGIIASLNARTSVLACANPTESRYNPRLSVID   613
AtMCM4               IDEFDKMSDSARSMLHEVMEQQTVSIAKAGIIASLNARTSVLACANPSGSRYNPRLSVIE   607
BoMCM4               IDEFDKMSDSARSMLHEVMEQQTVSIAKAGIIASLNARTSVLACANPSGSRYNPRLSVIE   585
BrMCM4               IDEFDKMSDSARSMLHEVMEQQTVSIAKAGIIASLNARTSVLACANPSGSRYNPRLSVIE   585
MCM4_Glyma11g12110   IDEFDKMSDNARSMLHEVMEQQTVSIAKAGIIASLNARTSVLACANPSGSRYNPRLSVID   597
MCM4_Glyma12g04320   IDEFDKMSDNARSMLHEVMEQQTVSIAKAGIIASLNARTSVLACANPSGSRYNPRLSVID   601
PsMCM4               IDEFDKMSDNARSMLHEVMEQQTVSIAKAGIIASLNARTSVLACANPSGSRYNPRLSVID   596
                     *******    :  : *.**:*:::*:**** : *::* :::*.***  . *:

BoMCM6_2             NVNLPPAILSRFDLVYL-------------------------------------------   438
BoMCM6_1             NVNLPPAILSRFDLVYVMIDDPDELTDYHIAHHIVRVHQKHEAA----------------   561
AtMCM6               NVNLPPAILSRFDLVYVMIDDPDEVTDYHIAHHIVRVHQKHEAA----------------   561
BrMCM6               NVNLPPAILSRFDLVYVMIDDPDELTDYHIAHHIVRVHQKHEAA----------------   561
ZmMCM6               NVALPPAILSRFDLVYIMIDEPDENTDYHIAHHIVRVHQKREEA----------------   565
PsMCM6               NVALPPAILSRFDLVYIMIDDPDDNTDYHIASHIVRVHQKREDA----------------   559
MCM6_Glyma09g05240   NVALPPAILSRFDLVYVMIDDPDDQTDYHIAHHIVRVHQKREGA----------------   560
MCM6_Glyma15g16570   NVALPPAILSRFDLVYVMIDDPDDQTDYHIAHHIVRVHQKREGA----------------   560
AtMCM2               NVELTDPILSRFDILCVVKDVVDPVTDEMLAEFVVNSHFKSQPK-GGKM-----------   719
BoMCM2_1             NVELTDPILSRFDILCVVKDVVDPVTDEMLAEFVVNSHFKSQPK-GGKM-----------   707
BrMCM2_1             NVELTDPILSRFDILCVVKDVVDPVTDDMLAEFVVNSHFKSQPK-GGKM-----------   710
BoMCM2_2             NVELTDPILSRFDILCVVKDVVDPVTDEMLAEFVVNSHFKSQPK-GGKM-----------   705
BrMCM2_2             NVELTDPILSRFDILCVVKDVGDPGTDEMLAEFVVNSHFKSQPK-GGKM-----------   714
ZmMCM2               NVELTDPIISRFDVLCVVKDIVDPFTDEMLARFVVDSHARSQPK-GANL-----------   740
PsMCM2               NVELTDPIISRFDILCVVKDVVDPVTDEMLAKFVVDSHFKSQPK-GANN-----------   720
MCM2_Glyma07g36680   NVELTDPIISRFDILCVVKDVVDPVTDEMLATFVVDSHFKSQPK-GANQ-----------   713
MCM2_Glyma17g03920   NVELTDPIISRFDILCVVKDVVDPVTDEMLATFVVDSHFKSQPK-GAKQ-----------   719
ZmMCM5               NIDLQTTILSRFDLIFIVKDIRMYDQDKRIASHIIKVHASGAAAS---------------   543
AtMCM5               NIDLQTTILSRFDLIFIVKDIRKYSQDKEIASHIIRVHASANKFS---------------   542
BoMCM5               NIDLQTTILSRFDLIFIVKDIRKYSQDKEIASHIIRVHASADKVT---------------   542
BrMCM5               NIDLQTTILSRFDLIFIVKDIRKYSQDKEIASHIIRVHASADKVT---------------   542
PsMCM5               NIDLQTTILSRFDLIFIVKDIRMYDQDKTIASHIIKVHASASATR---------------   545
MCM5_Glyma13g22420   NIDLQTTILSRFDLIFIVKDVRMYSQDKIIANHIIKVHKSAGGRM---------------   547
MCM5_Glyma17g11220   NIDLQTTILSRFDLIFIVKDVRMYSQDKIIANHIIKVHKSAGGRM---------------   547
ZmMCM3               NIGLPDSLLSRFDLLFIVLDQMDPEIDRQISEHVARMHRYCT--DDGGARSLDKEGYAEE   520
AtMCM3               NIGLPDSLLSRFDLLFIVLDQMDAGIDSMISEHVLRMHRYKN--DRGEAGPDGSLPYARE   515
BrMCM3_1             NIGLPDSLLSRFDLLFIVLDQMDAGIDSMISEHVLRMHRYQN--DRGEAGPDGNLPYGRD   517
BoMCM3               NIGLPDSLLSRFDLLFIVLDQMDAGIDSMISEHVLRMHRYQN--DRGEAGPDGNLPYGRE   517
```

```
BrMCM3_2              NIGLPDSLLSRFDLLFIVLDQMDAGIDSMISEHVLRMHRYQN--DRGEAGPDGNLPYGRE   517
MCM3_Glyma05g25980    NIGLPDSLLSRFDLLFIVLDQMDPDIDRRISEHVLRMHRFRSAVDGGEAVLHGSSRYGRE   521
MCM3_Glyma08g08920    NIGLPDSLLSRFDLLFIVLDQMDPDIDRRISEHVLRMHRFRSAVDGGEAALDGSSRYGRE   521
PsMCM3                NIGLPDSLLSRFDLLFIVLDQMDPDIDRQISEHVLRMHRFRSAIDGGEAAHDGSARYGKK   454
AtMCM7                NINLPPALLSRFDLLWLILDRADMDSDLELAKHVLHVHQTEESP----------------   542
BoMCM7_2              NINLPPALLSRFDLLWLILDRADMDSDLELAKHVLHVHQTQESP----------------   544
BrMCM7_1              NINLPPALLSRFDLLWLILDRADMDSDLELAKHVLHVHQTQESP----------------   544
BoMCM7_1              NINLPPALLSRFDLLWLILDRADMDSDLELAKHVLHVHQTHESP----------------   544
BrMCM7_2              NINLPPALLSRFDLLWLILDRADMDSDLELAKHVLHVHQTHESP----------------   537
ZmMCM7                NINLPPALLSRFDLLWLILDRADMETDLEMARHVVHVHQNLESP----------------   544
PsMCM7                NINLPPALLSRFDLLWLILDRADMDNDLEMARHVVYVHQNKESP----------------   544
MCM7_Glyma03g37770    NINLPHALLSRFDLLWLILDRADMDNDLEMARHVLYVHQNKESP----------------   544
MCM7_Glyma19g40370    NINLPHALLSRFDLLWLILDRADMDNDLEMARHVVYVHQNKESP----------------   544
ZmMCM4                NIHLAPTLLSRFDLIYLILDKADEQTDRRLAKHIVSLHFENPN-----------------   656
AtMCM4                NIHLPPTLLSRFDLIYLILDKPDEQTDRRLAKHIVALHFENAE-----------------   650
BoMCM4                NIHLPPTLLSRFDLIYLILDKPDEQTDRRLAKHIVALHFENAE-----------------   628
BrMCM4                NIHLPPTLLSRFDLIYLILDKPDEQTDRRLAKHIVALHFENAE-----------------   628
MCM4_Glyma11g12110    NIHLPPTLLSRFDLIYLMLDKADEQTDRRLAKHIVSLHFENPE-----------------   640
MCM4_Glyma12g04320    NIHLPPTLLSRFDLIYLMLDKAHEQTDRRLAKHIVSLHFENPE-----------------   644
PsMCM4                NIHLPPTLLSRFDLIYLLLDKADEQTDRRLAKHIVSLHFKDHE-----------------   639
                      *: *   ::****:: :

BoMCM6_2              -----------------------------------------------------SPEARKL   445
BoMCM6_1              ----------------------------LSPEFTTVQLKRYIAYAKT-LKPKLSPEARKL   592
AtMCM6                ----------------------------LSPEFTTVQLKRYIAYAKT-LKPKLSPEARKL   592
BrMCM6                ----------------------------LSPEFTTVQLKRYIAYAKT-LKPKLSPEARKL   592
ZmMCM6                ----------------------------LAPAFSTAQLKRYISFAKS-LKPQLSSEAKKV   596
PsMCM6                ----------------------------LAPTFTTAELKRYIAYAKT-LKPKLTSDARKL   590
MCM6_Glyma09g05240    ----------------------------LAPAFTTAELKRYIAYAKI-LKPKLSPDARKL   591
MCM6_Glyma15g16570    ----------------------------LAPAFTTAELKRYIAYAKT-LKPKLSPDARKL   591
AtMCM2                -----EDSD---PEDGIQG----SSGSTDPEVLPQNLLKKYLTYSKLYVFPKLGELDAKK   767
BoMCM2_1              -----DDSE---PQDDNHG----SSGSSDPEVLPQNLLRKYLTYSKLYVFPKLSEIDAKK   755
BrMCM2_1              -----DDSE---PQDDNHG----SSGSSDPEVLPQNLLRKYLTYSKLYVFPKLSEIDAKK   758
BoMCM2_2              -----DDSE---PQDVVQG----SSGSSDPEVLPQDLLRKYLTYSKLYVFPKLSEIDAKK   753
BrMCM2_2              -----DDSE---PQDGVQG----SSGSSDPEVLPQNLLRKYLTYSKLYVFPKLSEIDAKK   762
ZmMCM2                -----EDRVSTDVDDDPLA----AARQADPDVLSQDMLKKYITYAKLNVFPKIHDADLDK   791
PsMCM2                -----DDKSVSESQ-D--A----SGMPTDPEILPQDLLKKYSTYAKLNVFPRFNDVDLDK   768
MCM2_Glyma07g36680    -----DDKSFSESQ-DVHA----SAMPADPEILPQQLLKKYITYAKLNIFPRLQDADMDK   763
MCM2_Glyma17g03920    -----DDKSFSEFQ-DIHA----SAMPADPEILPQQLLKKYITYAKLNIFPRLQDADMDK   769
ZmMCM5                ------------------------ST-NTEGSEGENWLKRYIEYCRATCKPRLSEKAAEM   578
AtMCM5                ------------------------DENT--DSKEDNWLKRYIQYCRARCHPRLSKDAAEN   576
BoMCM5                ------------------------DENT--DSKEDNWLKRYIQYCRSRCHPRLTEAAAMK   576
BrMCM5                ------------------------DENT--DSKEDNWLKRYIQYCRSRCHPRLTEAAAMK   576
PsMCM5                ------------------------GENKTIISKEENWLKRYLKYCRTECHPRLSETAAKL   581
MCM5_Glyma13g22420    ------------------------GESRT--FKEENWLKRYLQYCRTQCHPRLSESATTL   581
MCM5_Glyma17g11220    ------------------------GESRT--LKEENWLKRYLQYCRTQCHPRLSESATTL   581
ZmMCM3                DDGDANAAIFVKYDRMLHGQDRRRGKKSKQDRLTVKFLKKYIHYAKNLIQPRLTDEASDH   580
AtMCM3                DN--AESEMFVKYNQTLHGKK-KRGQT-HDKTLTIKFLKKYIHYAKHRITPKLTDEASER   571
BrMCM3_1              DD--GESEVFVKYNRMLHGKKKKRGQT-NEKTLTIKFLKKYIHYAKHRIQPELTDEASER   574
BoMCM3                ED--GESEVFVKYNRMLHGKKKKRGQT-NEKTLTIKFLKKYIHYAKHRIQPELTDEASER   574
BrMCM3_2              ED--GESEVFVKYNRMLHGKKKKRGQT-NEKTLTIKFLKKYIHYAKHRIQPELTVEASER   574
MCM3_Glyma05g25980    DEADMDSSVFVKYNRMLHGKKTGRGQK-R-DTLTIKFLKKFIHYAKHRIQPELTDEASEN   579
MCM3_Glyma08g08920    DEADMDSSVFVKYNRMLHGKKTGRGQK-R-DTLTIKFLKKFIHYAKHRIQPELTDEASEN   579
PsMCM3                EEADTESSVFVKYNRMLHGKKTDRGRK-R-DTLTIKFLKKYIHYAKHRIQPDLTDEASDQ   512
AtMCM7                --------------------------ALGFEPLEPNILRAYISAARR-LSPYVPAELEEY   575
BoMCM7_2              --------------------------ALGFEPLEPNILRAYISAARR-LSPYVPAELEEY   577
BrMCM7_1              --------------------------ALGFEPLEPNILRAYISAARR-LSPYVPAELEEY   577
BoMCM7_1              --------------------------ALGFEPLEPNILRAYISAARR-LSPYVPAELEEY   577
BrMCM7_2              --------------------------ALGFDPLEPNILRAYISAARR-LSPYVPAELEEY   570
ZmMCM7                --------------------------ALGFTPLEPSVLRAYISAARR-VIPSVPRELEEY   577
PsMCM7                --------------------------ALGFTPLEPSVLRAYISTARR-LSPTVPRELEEY   577
```

```
MCM7_Glyma03g37770      --------------------------ALGFTPLEPSVLRAYISAARR-LSPSVPRELEEY    577
MCM7_Glyma19g40370      --------------------------ALGFTPLEPSVLRAYISAARR-LSPSVPRELEEY    577
ZmMCM4                  --------------------------LEELEVLDLQTLVSYISYARKYIQPQLSDEAAEE    690
AtMCM4                  --------------------------SAQEEAIDITTLTTYVSYARKNIHPKLSDEAAEE    684
BoMCM4                  --------------------------SAQEEALDITTLTSYVSYARKNIHPKLSDEAAEE    662
BrMCM4                  --------------------------SAQEEALDITTLTSYVSYARKNIHPKLSDEAAEE    662
MCM4_Glyma11g12110      --------------------------NVEQDVLDISTLTDYVSYARKHIHPQLSDEAAEE    674
MCM4_Glyma12g04320      --------------------------NVEQDVLDISTLTDYVSYARRHIHPQLSDEAAEE    678
PsMCM4                  --------------------------AMEQDVLDISTLTDYVSYARKHIHPQLSDEAADE    673


BoMCM6_2                LVDSYVDLRSGDTTP----GTRVAYRMTV----------------------KPSHVLLAV    479
BoMCM6_1                LVESYVALRRGDTTP----GTRVAYRMTVRQLEALIRLSEAIARSHLETLVKPSHVLLAV    648
AtMCM6                  LVESYVALRRGDTTP----GTRVAYRMTVRQLEALIRLSEAIARSHLEILVKPSHVLLAV    648
BrMCM6                  LVESYVALRRGDTTP----GTRVAYRMTVRQLEALIRLSEAIARSHLETLVKPSHVLLAV    648
ZmMCM6                  LVESYVTLRRGDSTP----GTRVAYRMTVRQLEALIRLSEAIARSHLERVVLPAHVRLAV    652
PsMCM6                  LVDSYVALRRADTNP----GSRVAYRMTVRQLEALIRLSEAIARCHLDNQVQPRHVRLAV    646
MCM6_Glyma09g05240      LVDSYVALRRGDTNP----GSRVAYRMTVRQLEALIRLSEAIARCHLDNEVQPRHVRLAV    647
MCM6_Glyma15g16570      LVDSYVALRRGDTNP----GSRVAYRMTVRQLEALIRLSEAIARCHLDNEVQPRHVRLAV    647
AtMCM2                  LETVYANLRRESMN-------GQGVSIATRHLESMIRMSEAHARMHLRQYVTEEDVNMAI    820
BoMCM2_1                LETVYANLRRESMN-------GQGVSIATRHLESMIRMSEANARMHLRQYVTEDDVNMAI    808
BrMCM2_1                LETVYANLRRESMN-------GQGVSIATRHLESMIRMSEANARMHLRQYVTEDDVNMAI    811
BoMCM2_2                LETVYANLRRESMN-------GQGVSIATRHLESMIRMSEAHARMHLRQYVTEEDVNMAI    806
BrMCM2_2                LETVYANLRRESMN-------GQGVSIATRHLESMIRMSEAHARMHLRQYVTEEDVNMAI    815
ZmMCM2                  ISHVYAELRRESSH-------GQGVPIAVRHIESIIRMSEAHARMHLRSYVSQEDVDMAI    844
PsMCM2                  LTHVYAELRKESSH-------GQGVPIAVRHIESMIRMSEAHARMHLRQHVTPEDVDMAI    821
MCM2_Glyma07g36680      LSHVYAELRRESSH-------GQGVPIAVRHIESMIRMSEAHARMHLRQHVTQEDVDMAI    816
MCM2_Glyma17g03920      LSHVYAELRRESSH-------GQGVPIAVRHIESMIRMSEAHARMHLRQHVTQEDVDMAI    822
ZmMCM5                  LQNKYIEIRQKMRQQAHETGRAAAIPITVRQLEAIIRLSESLAKMRLTSVATPEHVEEAF    638
AtMCM5                  LQRKYVTIRMDMKRRAHETGEAAPIPITVRQLEAIVRLSESLAKMRLSHEATPDDVDKAF    636
BoMCM5                  LQQQYVKIREDMKRRAHETGEAAPIPITVRQLEAIVRLSESLAKMRLSHDATEDDVGKAF    636
BrMCM5                  LQQQYVKIREDMKRRAHETGEAAPIPITVRQLEAIVRLSESLAKMRLSHDATEDDVGKAF    636
PsMCM5                  LQNNYVKIRQDMRQQANETGAAAAIPITVRQLEAIVRLSESLAKMKLSHLATEENVQEAI    641
MCM5_Glyma13g22420      LQNHYVKIRQDMRQQANETGEAAAIPITVRQLEAIVRLSEALAKMKLSHLATEENVQEAV    641
MCM5_Glyma17g11220      LQNHYVKIRQDMRQQANETGEAAAIPITVRQLEAIVRLSEALAKMKLSHLATEENVQEAV    641
ZmMCM3                  IATSYAELRDGSANA---KSGGGTLPITARTLESIIRLSTAHAKMKLRHEVLKSDVEAAL    637
AtMCM3                  IAEAYADLRNAGSDT---KT-GGTLPITARTLETIIRLATAHAKMKLSSEVTKADAEAAL    627
BrMCM3_1                IAEAYADLRNAGSDT---KT-GGTLPITARTLETIIRLATAHAKMKLSRKVTKADAEAAL    630
BoMCM3                  IAEAYADLRNAGSDT---KT-GGTLPITARTLETIIRLATAHAKLKLSRKVTKIDAEAAL    630
BrMCM3_2                IAEAYADLRNAGSDT---KT-GGTLPITARTLETIIRLATAHAKLKLSRKVTKTDAEAAL    630
MCM3_Glyma05g25980      IATAYAELRNASSNA---KT-GGTLPITARTLETIIRLSTAHAKLKLSREVSKSDVEAAL    635
MCM3_Glyma08g08920      IATAYAELRNSSSNA---KT-GGTLPITARTLETIIRLSTAHAKLKLSREVSKSDVEAAL    635
PsMCM3                  IATAYAELRNANSNA---KT-GGTLPITARTLETIIRLSTAHAKLKLSRKVTKSDVDAAL    568
AtMCM7                  IATAYSSIRQEEAKS-----NTPHSYTTVRTLLSILRISAALARLRFSESVAQSDVDEAL    630
BoMCM7_2                IATAYSSIRQEEAKS-----NTPHSYTTVRTLLSILRISAALARLRFSESVAQSDVDEAL    632
BrMCM7_1                IATAYSSIRQEEAKS-----NTPHSYTTVRTLLSILRISAALARLRFSESVAQSDVDEAL    632
BoMCM7_1                IATAYSSIRQEEAKS-----NTPHSYTTVRTLLSILRISAALARLRFSESVAQSDVDEAL    632
BrMCM7_2                IATAYSSIRQEEAKS-----NTPHSYTTVRTLLSILRISAALARLRFSESVAQSDVDEAL    625
ZmMCM7                  IATAYSSIRQEEAKS-----NAPTSYTTIRTLLSILRISIALARLRFSETVAQSDVDEAL    632
PsMCM7                  IASAYSSIRQEEAKS-----TTPHSYTTVRTLLSILRISAALARLRFAETVAQSDVDEAL    632
MCM7_Glyma03g37770      IATAYSSIRQEEARS-----NAPHSYTTVRTLLSILRISAALARLRFSETVAQSDVDEAL    632
MCM7_Glyma19g40370      IATAYSCIRQEEARS-----NAPHSYTTVRTLLSILRISAALARLRFSETVAQSDVDEAL    632
ZmMCM4                  LTRGYVEMRKRGNSP---GSRKKVITATARQIESLIRLSEALARMRFSEVVEVRDVVEAF    747
AtMCM4                  LTRGYVELRKAGKFA---GSSKKVITATPRQIESLIRLSEALARMRFSEWVEKHDVDEAF    741
BoMCM4                  LTRGYVELRKAGKFA---GSSKKVITATPRQIESLIRLSEALARMRFSEWVEKHDVEEAF    719
BrMCM4                  LTRGYVELRRAGKFA---GSSKKVITATPRQIESLIRLSEALARMRFSEWVEKDDVEEAF    719
MCM4_Glyma11g12110      LTRGYVEIRKRGNFP---GSSKKVITATPRQIESLIRLSEALARMRFSEWVEKHDVMEAF    731
MCM4_Glyma12g04320      LTRGYVEIRKRGNFP---GSSKKVITATPRQIESLIRLSEALARMRFSEWVEKHDVMEAF    735
PsMCM4                  LITGYVKIRGRGKFT---GSSKKVITATPRQIESLLRLSEALARIRFSEWVEKHDVLEAF    730
                        :   *  :*                  :                          ..  *.
```

```
BoMCM6_2             TLLKTSVISVESADVDLSEYQDANGDNIDDTDDVENTGNGDKEQQNGAA-----------   528
BoMCM6_1             RLLKTSVISVESGDIDLSEYQDANGDNMDNADDAENPVNGDEDQQNGSA-----------   697
AtMCM6               RLLKTSVISVESGDIDLSEYQDANGDNMDDTDDIENPVDGEEDQQNGAA-----------   697
BrMCM6               RLLKTSVISVESGDIDLSEYQDANGDNMDNADDADNPANGDEDQQNGSA-----------   697
ZmMCM6               KLLKTSIISVESSEVDLSDFQDAEDGTNVPSES-------DAGQPAEEDA-APQQQG---   701
PsMCM6               KLLQTSIIRVESSEIDLSEFQDQDREEEAGSGDGNNNN-NDADGTN--------------   691
MCM6_Glyma09g05240   KLLKTSIISVESSEIDLSEFQEENHDDGAGGGDGNDKN-RDANDQVGNDAAAQQAAGNA-   705
MCM6_Glyma15g16570   KLLKTSIISVESSEIDLSEFQEQNHDDGAGGGNENDNN-RDANYQEGNDTAQ-QAAGNG-   704
AtMCM2               RVLLDSFISTQKFGVQRTLRESFKRYIT-------------YKKD---------F-----   853
BoMCM2_1             RVLLDSFISTQKFGVQRTLRESFKRYIT-------------YKKD---------Y-----   841
BrMCM2_1             RVLLDSFISTQKFGVQRTLRESFKRYIT-------------YKKD---------Y-----   844
BoMCM2_2             RVLLDSFISTQKFGVQRTLRESFKRYIT-------------YKKD---------Y-----   839
BrMCM2_2             RVLLDSFISTQKFGVQRTLRESFKRYIT-------------YKKD---------Y-----   848
ZmMCM2               RVLLDSFISTQKFGVQKALQKNFRKYMT-------------FKKD---------Y-----   877
PsMCM2               RVLLDSFISTQKFGVQKALQKSFRKYIT-------------FKKD---------Y-----   854
MCM2_Glyma07g36680   RVLLESFISTQKFGVQKALQKSFRKYMT-------------FKKD---------Y-----   849
MCM2_Glyma17g03920   RVLLESFISTQKFGVQKALQKSFRKYMT-------------FKKD---------Y-----   855
ZmMCM5               RLFNVSTVDAARSGINEHLNLSPEIANEIKQAEAQIKRRMGIGSH--------------I   684
AtMCM5               KLFDTSTMDAARSGINQQINITGEMANEIKQAETQIKRRMGIGAR--------------L   682
BoMCM5               KLFDTSTMDAARSGINQQINITSEMANEIKQAETQIKRRMGIGAR--------------L   682
BrMCM5               KLFDTSTMDAARSGINQQINITSEMANEIKQAETQIKRRMGIGAR--------------L   682
PsMCM5               RLFTVSTMDAAKSGINQQINLTPEMAHEIQQAEIQIKRRIGIGNH--------------I   687
MCM5_Glyma13g22420   RLFTVSTMDAAKSGINQQINLTPDMANEIKQAETQIKRRIGIGNH--------------I   687
MCM5_Glyma17g11220   RLFTVSTMDAAKSGINQQINLTPDMANEIKQAETQIKRRIGIGNH--------------I   687
ZmMCM3               QVLNFAIYHKELTEMEEREQKEMEMK----QQAEH-----DAGA----------TGGTVD   678
AtMCM3               KLMNFAIYHQELTEMDDREQEERQRE----QAEQERTPSGRRGNQRRNN--EDGAENDTA   681
BrMCM3_1             KLMNFAIYHQELTEMDEREQEERQRE----QTEQERTPRG---NQRRSD--DNEQENVSA   681
BoMCM3               KLMNFAIYHQELTEMDEREQEERQRE----QSEQERTPSG---HR-------GNQENVSV   676
BrMCM3_2             KLMNFAIYHQELTEMDEREQEERQRE----QSEQERTPSG---HR-------GNQENVSV   676
MCM3_Glyma05g25980   KVLNFAIYHKELTEMEEREQERERERL----DR----KRKADHDENDGPDHGPKDRRGPKD   687
MCM3_Glyma08g08920   KVLNFAIYHKELTEMEEREQEREREL----DR----KRKADHDENDGPDRGPKDRRGPKD   687
PsMCM3               KILNFAIYHKELTEMDEREEEEREKER----EREQERKRKA-DAENNGPDRGSKSK-----   618
AtMCM7               RLMQMSKISLYADDRQ-KAGLDAISDTY-SIIRDEAARSKKTHVS---------Y-----   674
BoMCM7_2             RLMQMSKISLYADDRQ-KAGLDAISDTY-SIIRDEAARSNKTHVS---------Y-----   676
BrMCM7_1             RLMQMSKISLYADDRQ-KAGLDAISDTY-SIIRDEAARSNKTHVS---------Y-----   676
BoMCM7_1             RLMQMSKISLYVDDRQ-KAGLDAISDTY-SIIRDEAARSNKTHVS---------Y-----   676
BrMCM7_2             RLMQMSKISLYADDRQ-KAGLDAISDTY-SIIRDEAARSNKTHVS---------Y-----   669
ZmMCM7               RLMQMSKYSLYSDDRQ-RSGLDAISDIY-SILRDEAARTSSMDVR---------Y-----   676
PsMCM7               RLMQMSKFSLYSDDRQ-RSGLDAISDIY-SILRDEAARSHKMDVN---------Y-----   676
MCM7_Glyma03g37770   RLMQMSKFSLYSEDRQ-KSGLDAISDIY-SILRDEAARGNRMDVS---------Y-----   676
MCM7_Glyma19g40370   RLMQMSKFSLYSEDRQ-KSGLDAISDIY-SILRDEAARGNRMDVS---------Y-----   676
ZmMCM4               RLLEVAMQQSATDHATGTIDMDLIMTGI---SASERQRRENLVAA---------T-----   790
AtMCM4               RLLRVAMQQSATDHATGTIDMDLINTGV---SASERMRRDTFASS---------I-----   784
BoMCM4               RLLRVAMQQSATDHATGTIDMDLINTGV---SASERMRRDILASS---------I-----   762
BrMCM4               RLLRVAMQQSATDHATGTIDMDLINTGV---SASERMRRDILVSS---------I-----   762
MCM4_Glyma11g12110   RLLEVAMQQSATDHSTGTIDMDLITTGV---SASERMRRESLQQA---------T-----   774
MCM4_Glyma12g04320   RLLEVAMQQSATDHSTGTIDMDLITTGV---SASERMRRESLQQA---------T-----   778
PsMCM4               RLLEVAMQQSAMDIKTGTIDMDLITTGV---SASERIRRESLIQD---------T-----   773
                      ::  :

BoMCM6_2             ------------------------EPA---SAT-ADKGAVAPKLVINEEEYDKITQALVI   560
BoMCM6_1             ------------------------EPA---PATAADNGAAAPKLVISEEEYDRITQALVL   730
AtMCM6               ------------------------EPA---SAT-ADNGAAAQKLVISEEEYDRITQALVI   729
BrMCM6               ------------------------EAA---PAT-ADNGAAAPKLVISEEEYDRITQALVL   729
ZmMCM6               ---------------------------AENDQ---AADNGKKKLVITEEHFQRVTQALVM   731
PsMCM6               ---------------------------GDNEKAADESNPQRKKSTVTDEYFQRITRALVT   724
MCM6_Glyma09g05240   -NDQVGNDATQQTANDQVGNDAAQQPAGNNGNSADGSKPQVRKLVMSDEYYQRVTSALIM   764
MCM6_Glyma15g16570   --------------NDQVGNDAAQQATGNNGNPADGSKPQVRKLIMSDEYYQRVTSALIM   750
AtMCM2               --NSLLLVLL------KEL---------VKNALKF---------EEIISGS--NSGLPTI   885
BoMCM2_1             --NSLLLVLL------KEL---------VKNALKF---------EEIITGS--NSGLSSI   873
BrMCM2_1             --NSLLLVLL------KEL---------VKNALKF---------EEIITGS--NSGLSSI   876
```

```
BoMCM2_2              --NSLLLVLL------KEL---------VKNAMKF---------EEIITGS--NSGLPSI   871
BrMCM2_2              --NSLLLVLL------KEL---------VKNAMKF---------EEIITGS--NSGLPFI   880
ZmMCM2                --NELLLLLL------RTL---------VKDAVHF---------EEIMAGS--ASRLTHV   909
PsMCM2                --NDMLIYIL------QEL---------VKSAIKF---------EEIVAGS--TSSLTHV   886
MCM2_Glyma07g36680    --NELLLYIL------REL---------VKNALHF---------EEIVTGS--ASGLTHI   881
MCM2_Glyma17g03920    --NELLLYIL------REL---------VKNALHF---------EEIVTGS--ASGLTHI   887
ZmMCM5                SERRLIDDLN------RMG---------MNES---------------IVRR---------   705
AtMCM5                SERRLIEDLA------RMG---------MNDS---------------MVRR---------   703
BoMCM5                SERRLIEDLA------RMG---------MNDS---------------MVRR---------   703
BrMCM5                SERRLIEDLA------RMG---------MNDS---------------MVRR---------   703
PsMCM5                SERRLIDDLG------RMG---------MNDS---------------IVRR---------   708
MCM5_Glyma13g22420    SERRLIDDLS------RMG---------MNES---------------IVRR---------   708
MCM5_Glyma17g11220    SERRLIDDLS------RMG---------MNES---------------IVRR---------   708
ZmMCM3                GHGSSGNDPM------DVD---------VGSN---DQ-NV---SAERIEAFEALLGQHVL   716
AtMCM3                NVDSETADPM------EVD---------EPSVEQFSG-TV---SAARIETFERVFGQHMR   722
BrMCM3_1              NAESETADPM------DVD---------EPPAEQFSG-TV---SAARIETFERVFGQHMR   722
BoMCM3                NVESETADPM------DVD---------EPPSEQFSG-TV---SAARIESFERVFGQHMR   717
BrMCM3_2              NVESETADPM------DVD---------EPPSEQFSG-TV---SAARIESFERVFGQHMR   717
MCM3_Glyma05g25980    KRRSTSTDAM------DVD---------DNSAAQAAV-GL---TPERIEEFNSLFNQHMH   728
MCM3_Glyma08g08920    KRGPTSTDAM------EVD---------DNSATHAAV-GP---TPERIEEFNSLFNQHMH   728
PsMCM3                --RDSTSEAM------ELD---------DTSAAPPAV-GL---TPERIEAFNSLFGQHMR   657
AtMCM7                --ANALNWIS------RKG---------YS------E-AQ---LKECLEEYAALN---VW   704
BoMCM7_2              --ANALNWIS------RKG---------YS------E-AQ---LKECLEEYAALN---VW   706
BrMCM7_1              --ANALNWIS------RKG---------YS------E-AQ---LKECLEEYAALN---VW   706
BoMCM7_1              --ANALNWIS------RKG---------YS------E-AQ---LKECLEEYAALN---VW   706
BrMCM7_2              --ANALNWIS------RKG---------YS------E-AQ---LKECLEEYAALN---VW   699
ZmMCM7                --AHALNLIS------RKG---------YS------E-AQ---LKECLEEYASLN---VW   706
PsMCM7                --RDALNWIS------RKG---------YS------E-AQ---LKECLEEYAALN---VW   706
MCM7_Glyma03g37770    --AHALNWIS------RKG---------YS------E-AQ---LKECLEEYAALN---VW   706
MCM7_Glyma19g40370    --AHALNWIS------RKG---------YS------E-AQ---LKECLEEYAALN---VW   706
ZmMCM4                --RNLIAEKM------QLG---------GP------S-MR---MIELLEELRKQS---SM   820
AtMCM4                --RDIALEKM------QIG---------GS------S-MR---LSELLEELKKHGGNINT   817
BoMCM4                --RDITLEKM------QIG---------GS------A-MR---LSELLEELKKHGGNINT   795
BrMCM4                --RDITLEKM------QIG---------GS------A-MR---LSELLEELKKHGGNINT   795
MCM4_Glyma11g12110    --RNIIMEKM------QIG---------GP------S-MR---LLELLEELKKQD--TGS   805
MCM4_Glyma12g04320    --RNIIMEKM------QIG---------GQ------S-MR---LLELLEELKKQD--TGS   809
PsMCM4                --RNIILEKM------QIG---------GR------S-MR---LLEILEELKNQS--PGN   804


BoMCM6_2              RLRQHEETVS-----------------KDSSELPGMR-------QKELIRWYIDQQNEKK   596
BoMCM6_1              RLRQHEETVN-----------------KDSSELPGMR-------QKELIRWYIDQQNEKK   766
AtMCM6                RLRQHEETVN-----------------KDSSELPGIR-------QKELIRWFIDQQNEKK   765
BrMCM6                RLRQHEETVK-----------------KDCSELPGMR-------QKELIRWYIDQQNEKK   765
ZmMCM6                RLRQHEESVK-----------------KDGDGLAGMK-------QGDLIIWYVEQQNAKG   767
PsMCM6                RLRQHEETVV-----------------EQGSDLAGMR-------QRDLIKWYVDQQNEKN   760
MCM6_Glyma09g05240    RLRQHEEAVV------------------QGNGLSGMR-------QKDLIQWYVDQQNERN   799
MCM6_Glyma15g16570    RLRQHEEAVV------------------QGDGLSGMR-------QKDLIQWYVDQQNERN   785
AtMCM2                EVKIEELQTKAKEYDIADLRPFFSSTDFSKAHFELDHGRGMIKCPKRLITW---------   936
BoMCM2_1              EVKIEELQTKANEYDLADLRPFFSSTDFAKAHFELDHGLGVIKFPRRLVTW---------   924
BrMCM2_1              KVKIEELQTKANEYDLADLRPFFSSKDFAKAHFELDHGLGVIKFPRRLVTW---------   927
BoMCM2_2              EVKIEELQTKANEYDIADLRPFFSSTDFAKAHFELDQVLGVIKCPRRLVTW---------   922
BrMCM2_2              EVKIEELQTKANEYDIADLRPFFSSTDFAKARFELDQVLGVIKCPRRLVTW---------   931
ZmMCM2                EVKLEDLRNKAQEYEIYDLKPFFSSACFRDNGFLLDEGRGIIRHPLAE------------   957
PsMCM2                EVKVDDLFVKAQEHDIYDLKPFFNSSQFSKSNYILDEERAVIRHNLV-------------   933
MCM2_Glyma07g36680    DVKVDDLYNKAQEHDIYDLKPFFNSSHFSRANFVLDEERRVIRHHLTR*-----------   929
MCM2_Glyma17g03920    DVKVDDLYHKAQEHDIYDLKPFFNSSHFSRANFVLDEERRVIRHHLTR*-----------   935
ZmMCM5                ---------A----------------------LLIMHQRDEVEYKRER------------   722
AtMCM5                ---------A----------------------LLIMHQRGEVEYQRER------------   720
BoMCM5                ---------A----------------------LLIMHQRGEVEYQRER------------   720
BrMCM5                ---------A----------------------LLIMHQRGEVEYQRER------------   720
PsMCM5                ---------A----------------------LLIMHQRDEVEYKRER------------   725
```

```
MCM5_Glyma13g22420      ---------A----------------------LIIMHQRDEIEYKRER------------    725
MCM5_Glyma17g11220      ---------A----------------------LIIMHQRDEIEYKRER------------    725
ZmMCM3                  ANHIDQMSID------------------EIEQMVNREST--APYTRSQVEFILERMQDAN    756
AtMCM3                  THRLDDISIA------------------DIETVVNNNGVGASRYSADEIMALLEKLQDDN    764
BrMCM3_1                TNRLDDISIA------------------DIETVVNNNGVGASRYSADEIMALLEKLQDDN    764
BoMCM3                  TNRLDDISIA------------------DIETVVNNNGVGASRYSADEIMALLEKLQDDN    759
BrMCM3_2                TNRLDDISIA------------------DIETVVNNNGVGASRYSADEIMALLEKLQDDN    759
MCM3_Glyma05g25980      ANRLDQITVV------------------NLVNVIN-RGEDPPSYSAADVLLLLERLQDDN    769
MCM3_Glyma08g08920      ANRLEQITIA------------------NLGNVIN-RGQDPPPYSAADILLLLERLQDDN    769
PsMCM3                  ANPLDQISIA------------------DIEDVIN-RGADS-TYSSADILLLLEKLQEDN    697
AtMCM7                  QIDPHTFDIR----------------------FI--------------------------    716
BoMCM7_2                QIDPNTFDIR----------------------FI--------------------------    718
BrMCM7_1                QIDPNTFDIR----------------------FI--------------------------    718
BoMCM7_1                QIDPNTFDIR----------------------FI--------------------------    718
BrMCM7_2                QIDPNTFDIR----------------------FI--------------------------    711
ZmMCM7                  QIHPSTFDIH----------------------FIDA------------------------    720
PsMCM7                  QIHPQTFDIK----------------------FIDA------------------------    720
MCM7_Glyma03g37770      QIHPHTFDIR----------------------FIDA*-----------------------    720
MCM7_Glyma19g40370      QIHPHTFDIR----------------------FIDA*-----------------------    720
ZmMCM4                  EIHMHELRGA--------------LGTLMTEGAVVIHGDNVRRV----------------    850
AtMCM4                  EIHLHDVRKA--------------VATLASEGFLVAEGDRIKRV----------------    847
BoMCM4                  EIHLHDVRKA--------------VGTLASEGFLVTEGDRIKRI----------------    825
BrMCM4                  EIHLHDVRKA--------------VGTLASEGFLVTEGDRIKRI----------------    825
MCM4_Glyma11g12110      EVHLNDLRNA--------------IATLATEGFVTMHGDIVKRS*---------------    835
MCM4_Glyma12g04320      DVHLNDLRNA--------------IATLAAEGFVTMHGDSVKRS*---------------    839
PsMCM4                  EIHLNDLRNA--------------VSTLASEGFLSVAGESVKRT----------------    834


BoMCM6_2                KYSSQEQVKLDIKKLRAIIESFVCKEGHLIVLANEQEEATEAEE-TRRKSSQRDERILAV    655
BoMCM6_1                KYTSQEQVKLDIKKLRAIIECYPMSKKPQKVKNQEEEMRG----------------SWPL    810
AtMCM6                  KYSSQEQVKLDIKKLRAIIESLVCKEGHLIVLANEQEEAAEAEE-TKKKSSQRDERILAV    824
BrMCM6                  KYTSQEQVKLDIKKLRAIIESLVCKEGHLIVLSNEQEAEGE-------EPRRRDERILAV    818
ZmMCM6                  AYSSTAEVKEEVKCIKAIIERLIQREGHLIVIDEGTAAAAEDGSGA---RRTSESRILAV    824
PsMCM6                  NYSSIEEAKTEVSQIKAIIEILIRREGHLIVVDDGRQAAAEAAGAEQTESAARNDRILAV    820
MCM6_Glyma09g05240      NYSSMEEVQAEISKIKAIIESLIRREGHLIVVDDGQAAAAAA----EPPGAPRNYRILAV    855
MCM6_Glyma15g16570      NYSSMDEVQAEISKIKAIIESLIRREGHLIVVDNGEAAAAAA----EPPGAPRNYRILAV    841
AtMCM2                  ------------------------------------------------------------    936
BoMCM2_1                ------------------------------------------------------------    924
BrMCM2_1                ------------------------------------------------------------    927
BoMCM2_2                ------------------------------------------------------------    922
BrMCM2_2                ------------------------------------------------------------    931
ZmMCM2                  ------------------------------------------------------------    957
PsMCM2                  ------------------------------------------------------------    933
MCM2_Glyma07g36680      ------------------------------------------------------------    929
MCM2_Glyma17g03920      ------------------------------------------------------------    935
ZmMCM5                  HVI--------------------VRKA---------------------------------    729
AtMCM5                  RSI--------------------VRKA---------------------------------    727
BoMCM5                  RSI--------------------VRKA---------------------------------    727
BrMCM5                  RSI--------------------VRKA---------------------------------    727
PsMCM5                  RVL--------------------FRKA---------------------------------    732
MCM5_Glyma13g22420      RVV--------------------FRKA*--------------------------------    732
MCM5_Glyma17g11220      RVV--------------------FRKA*--------------------------------    732
ZmMCM3                  RVM--------------------IRDGVVRII----------------------------    768
AtMCM3                  KVM--------------------ISDGKVHII----------------------------    776
BrMCM3_1                KVM--------------------ISDGKVHII----------------------------    776
BoMCM3                  KVM--------------------ISDGKVHII----------------------------    771
BrMCM3_2                KVM--------------------ISDGKVHII----------------------------    771
MCM3_Glyma05g25980      RVM--------------------ITDGVVHMIS*--------------------------    782
MCM3_Glyma08g08920      RVM--------------------ITDGVVHMIS*--------------------------    782
PsMCM3                  RLM--------------------IVAGMVHMIS---------------------------    710
AtMCM7                  ------------------------------------------------------------    716
```

```
BoMCM7_2            ------------------------------------------------------------    718
BrMCM7_1            ------------------------------------------------------------    718
BoMCM7_1            ------------------------------------------------------------    718
BrMCM7_2            ------------------------------------------------------------    711
ZmMCM7              ------------------------------------------------------------    720
PsMCM7              ------------------------------------------------------------    720
MCM7_Glyma03g37770  ------------------------------------------------------------    720
MCM7_Glyma19g40370  ------------------------------------------------------------    720
ZmMCM4              ------------------------------------------------------------    850
AtMCM4              ------------------------------------------------------------    847
BoMCM4              ------------------------------------------------------------    825
BrMCM4              ------------------------------------------------------------    825
MCM4_Glyma11g12110  ------------------------------------------------------------    835
MCM4_Glyma12g04320  ------------------------------------------------------------    839
PsMCM4              ------------------------------------------------------------    834


BoMCM6_2            ASNYVTE-        662
BoMCM6_1            LPTM----        814
AtMCM6              APNYVIE-        831
BrMCM6              APNYVIE-        825
ZmMCM6              NPNYVID-        831
PsMCM6              APHYVVD-        827
MCM6_Glyma09g05240  APNYVID*        862
MCM6_Glyma15g16570  APNYVID*        848
AtMCM2              --------        936
BoMCM2_1            --------        924
BrMCM2_1            --------        927
BoMCM2_2            --------        922
BrMCM2_2            --------        931
ZmMCM2              --------        957
PsMCM2              --------        933
MCM2_Glyma07g36680  --------        929
MCM2_Glyma17g03920  --------        935
ZmMCM5              --------        729
AtMCM5              --------        727
BoMCM5              --------        727
BrMCM5              --------        727
PsMCM5              --------        732
MCM5_Glyma13g22420  --------        732
MCM5_Glyma17g11220  --------        732
ZmMCM3              --------        768
AtMCM3              --------        776
BrMCM3_1            --------        776
BoMCM3              --------        771
BrMCM3_2            --------        771
MCM3_Glyma05g25980  --------        782
MCM3_Glyma08g08920  --------        782
PsMCM3              --------        710
AtMCM7              --------        716
BoMCM7_2            --------        718
BrMCM7_1            --------        718
BoMCM7_1            --------        718
BrMCM7_2            --------        711
ZmMCM7              --------        720
PsMCM7              --------        720
MCM7_Glyma03g37770  --------        720
MCM7_Glyma19g40370  --------        720
ZmMCM4              --------        850
AtMCM4              --------        847
BoMCM4              --------        825
```

```
BrMCM4                  ---------       825
MCM4_Glyma11g12110      ---------       835
MCM4_Glyma12g04320      ---------       839
PsMCM4                  ---------       834
```
